# Investigation of neuromodulation of the endbulb of Held synapse in the cochlear nucleus by serotonin and norepinephrine

**DOI:** 10.3389/fncel.2025.1575158

**Published:** 2025-04-28

**Authors:** Maria Groshkova, Theocharis Alvanos, Yumeng Qi, Fangfang Wang, Carolin Wichmann, Yunfeng Hua, Tobias Moser

**Affiliations:** ^1^Institute for Auditory Neuroscience and InnerEarLab, University Medical Center Göttingen, Göttingen, Germany; ^2^Auditory Neuroscience & Synaptic Nanophysiology Group, Max Planck Institute of Multidisciplinary Sciences, Fassberg Campus, Göttingen, Germany; ^3^Collaborative Research Center SFB 1286 “Quantitative Synaptology”, University of Göttingen, Göttingen, Germany; ^4^Göttingen Graduate School for Neurosciences and Molecular Biosciences, University of Göttingen, Göttingen, Germany; ^5^International Max Planck Research School for Molecular Biology (IMPRS), Göttingen, Germany; ^6^Shanghai Institute of Precision Medicine, Shanghai Ninth People's Hospital, Shanghai Jiao Tong University School of Medicine, Shanghai, China; ^7^Molecular Architecture of Synapses Group, Institute for Auditory Neuroscience and Center for Biostructural Imaging of Neurodegeneration, University Medical Center Göttingen, Göttingen, Germany; ^8^Multiscale Bioimaging Cluster of Excellence (MBExC), University of Göttingen, Göttingen, Germany

**Keywords:** neuromodulation, serotonin receptor, adrenergic receptor, synaptic plasticity, voltage-gated ion channels, excitability, cochlear nucleus

## Abstract

**Introduction:**

Synapses vary greatly in synaptic strength and plasticity, even within the same circuitry or set of pre- and postsynaptic neurons. Neuromodulation is a candidate mechanism to explain some of this variability. Neuromodulators such as monoamines can differentially regulate presynaptic function and neuronal excitability. Variability is found also for the large calyceal synapses of the auditory pathway that display high synaptic vesicle (SV) release probability (P_vr_) and large postsynaptic currents *in vitro* enabling reliable and temporally precise transmission of auditory information. In this study, we investigated whether the endbulb of Held synapse formed by auditory nerve fibers onto bushy cells (BCs) in the anteroventral cochlear nucleus (AVCN) of mice is modulated by norepinephrine (NE) and serotonin (5-HT).

**Methods:**

We used electron microscopy (EM) of the cochlear nucleus (CN) to investigate the presence of monoaminergic projections. Furthermore, we performed immunohistochemistry to study the localization of monoamine transporters and receptors in the AVCN. We performed patch-clamp recordings from BCs to study spontaneous and evoked synaptic transmission as well as short-term plasticity of the endbulb of Held synapse and to investigate the excitability of the BCs.

**Results:**

We found EM evidence for putative monoaminergic varicosities in both ventral and dorsal divisions of the CN. Immunostaining for vesicular 5-HT and NE transporters revealed NE-containing and 5-HT-containing varicosities in the AVCN, juxtaposed to both endbulbs and BCs. Furthermore, we detected immunofluorescence for 5-HT_1B_, 5-HT_4_, and 5-HT_7_ receptors (R) and α_2C_-adrenergic receptors (AR) in BCs. Patch-clamp recordings from BCs revealed an increase in frequency of miniature excitatory postsynaptic currents (mEPSCs) upon application of NE but not 5-HT. Evoked synaptic transmission was unaffected by the application of either NE or 5-HT. Similarly, when studying the biophysical properties of the BCs, we did not observe effects of NE or 5-HT on low-voltage-activated K^+^ (${\text{K}}_{\text{LVA}}^{+}$) and hyperpolarization-activated mixed cation (HCN) channels during application.

**Discussion:**

In summary, we report evidence for the presence of monoaminergic innervation in the cochlear nucleus and for subtle functional NE-neuromodulation at the endbulb of Held synapse.

## 1 Introduction

The endbulb synapse is characterized by synchronous release from dozens of active zones (AZs) for producing strong excitatory postsynaptic currents (EPSCs) for reliable and temporally precise transmission (Wang and Manis, 2005; Wang et al., 2010). In this way, this synapse enables a faithful representation of sound's temporal properties into the central auditory system and allows the performance of tasks such as encoding the onset of sound (Rhode, 2008) and sound localization (Kuenzel, 2019). A recent serial block face scanning EM (SBEM) study in the mouse AVCN described endbulbs, variable in size converging onto the same globular BC (Spirou et al., 2023). Based on compartmental modeling, the study suggested that variability in synaptic weights of different inputs contributed to improving the temporal precision of the neural code, leaving the AVCN that had been reported (Joris et al., 1994).

Aside from strong glutamatergic axosomatic input from the endbulbs formed by the spiral ganglion neurons (SGNs), temporally precise firing of BCs capitalizes on (I) fast rapidly desensitizing α-amino-3-hydroxy-5-methyl-4-isoxazolepropionic acid (AMPA) receptors (Isaacson and Walmsley, 1996; Yang and Xu-Friedman, 2008) and (II) short membrane time constant (τ_m_), which limits the time window of integration (Kuenzel, 2019). The fast τ_m_ corresponds to low input resistance, reflecting the presence of a low-voltage-activated potassium conductance (g_KL_) in BCs. The low-voltage-activated potassium channels (${\text{K}}_{\text{LVA}}^{+}$) mediating g_KL_ activate near the resting potential, producing large hyperpolarizing currents that prevent BCs from repetitive firing (Golding and Oertel, 2012). Furthermore, g_KL_ makes BCs sensitive to the rate of depolarization, by suppressing slow membrane depolarizations. An opposing hyperpolarization-activated cation conductance (g_h_), mediated by hyperpolarization and cyclic nucleotide activated (HCN) channels, is activated when BCs are exposed to strong hyperpolarizing currents. In BC, there is evidence for fast gating HCN1 channels (Oertel et al., 2008). This conductance shortens the refractory period of the BCs, enabling them to receive and respond to high frequency repetitive stimulation from SGNs (Cao et al., 2007). Hence, both g_KL_ and g_h_ define the time window of integration of signals in BCs and their ability to extract the temporal properties of SGN input.

While being tuned to reliable and precise transmission of auditory information, evidence for neuromodulation in the cochlear nucleus (CN) has been presented. Neuromodulators are molecules that can modify synaptic transmission and the excitability of the postsynaptic cell by affecting the properties of SV release, neurotransmitter receptors, or voltage-gated ion channels (Brzosko et al., 2019; Özçete et al., 2024). This effect is achieved through the activity of G protein-coupled receptors (GPCRs) resulting in second messenger cascades of signaling. In fact, a number of G-protein-modulated signal pathways have been shown to influence neurotransmitter release (de Jong and Verhage, 2009). Cholinergic transmission has been described to have an excitatory effect on BC (Goyer et al., 2016). Evidence for modulation was shown for the auditory pathway where calyceal synapses undergo GABA_B_ modulation (Takahashi et al., 1998; Brenowitz and Trussell, 2001). Modulation of presynaptic Ca^2+^ currents (Takahashi et al., 1998; Kimura et al., 2003) is probably the most effective way to influence neurotransmitter release due to the supralinear relationship between release and intracellular [Ca^2+^]. However, several modulators act via G_q_ signaling coupled to the phospholipase C (PLC)–diacylglycerol (DAG)–inositol 1,4,5-trisphosphate (IP_3_) pathway. The importance of the IP_3_ pathway as a target of neuromodulation of basal Ca^2+^ has not yet been demonstrated. In dendrites, the endoplasmic reticulum (ER) contacts the plasma membrane in regularly interspersed domains where the IP_3_ pathway forms local Ca^2+^ signaling hubs (Benedetti et al., 2025; periodic ER-plasma membrane junctions support long-range Ca^2+^ signal integration in dendrites). Such activity has not yet been shown for axonal or presynaptic compartments to our knowledge. The DAG pathway, for which the priming protein Munc13 is a prominent target (Rhee et al., 2002), is better poised to play a role in presynaptic compartments. Although it has been known for decades that phorbol ester, a mimic of DAG, increases synaptic strength by factors of 2 to 6 (e.g., Hori et al., 1999; Lou et al., 2005; Lee et al., 2013), little attention has been paid so far to the possibility of neuromodulation via this route. Furthermore, G_s_ signaling has been described for the calyx of Held that increased both the readily releasable pool and the release probability at the calyx of Held (Sakaba and Neher, 2001; Kaneko and Takahashi, 2004). In addition, NE modulation of the calyx of Held has been described to facilitate high frequency firing during development through Gq signaling (Leão and Von Gersdorff, 2002). Candidate neuromodulators of the CN include the monoamines dopamine, 5-HT, and NE. Monoamine modulators are mainly secreted in targeted areas in a process called volume transmission (VT) (Agnati et al., 1986; Fuxe and Borroto-Escuela, 2016). In this process, the neurotransmitter molecules diffuse through the extracellular fluid and interact with their respective neuromodulator receptors. The projections of neuromodulators-releasing neurons are known to form swellings along their length known as varicosities where the secretion is known to occur from (Séguéla et al., 1990; Descarries et al., 1996; Gianni and Pasqualetti, 2023). These typically unmyelinated swellings contain clear and dense core vesicles and most often lack classical synaptic specializations (Séguéla et al., 1990; Descarries and Mechawar, 2000; Liu et al., 2018). The concentration gradient that results from volume transmission seems to form domains of affinity, corresponding to a non-random compartmental placement of respective modulator receptors in the postsynaptic cells (Özçete et al., 2024).

Noradrenergic synaptic transmission takes part in processes such as arousal, attention, cognition, fear conditioning and memory formation (Groch et al., 2011). NE acts through two broad families of receptors, namely, α- and β-adrenergic receptors (ARs). The α-AR family is subdivided to α_1_-ARs, coupled with G_q_ proteins, activating PLC (Wu et al., 1992), leading to an increase of IP_3_ and intracellular Ca^2+^; and α_2_-ARs, acting through G_i/o_ proteins, thus lowering cyclic adenosine monophosphate (cAMP) levels by inhibiting adenylate cyclase (AC). Particularly, α_2_-AR signaling inhibited HCN currents in neurons in the prefrontal cortex (Carr et al., 2007; Wang et al., 2007; Zhang et al., 2013). Furthermore, activation of α_2_-ARs at climbing fibers in the cerebellum decreased P_vr_ and subsequently modulates short-term associative plasticity of the parallel fiber to Purkinje cell synapse (Carey and Regehr, 2009). NE differentially modulated short-term depression of evoked inhibitory transmission at layers I vs. layers II/III in the auditory cortex at 20 Hz stimulation frequency (Salgado et al., 2011). While its effect on layer I was to alter the depressive pattern to a facilitating one, in layers II/III the depression was even more prominent after NE application. In addition, one of the early findings on presynaptic neuromodulation was the inhibitory effect of NE on the release from cholinergic projections in the ileum longitudinal muscle strip of guinea pigs, mediated by α_2_-ARs (Paton and Vizi, 1969). In stem-cell-derived human neurons, the effect of α_2_-AR activation resulted in cAMP reduction and synapsin-1 de-phosphorylation. This augmented the reserve pool of SVs, countering the 5HT_7_R effect (see below) and allowing for bi-directional control of SV replenishment (Patzke et al., 2019). The β-AR family consists of three subgroups—β_1_-ARs, β_2_-ARs, and β_3_-ARs all acting through G_s_ proteins and elevating cAMP levels (Sibley and Lefkowitz, 1987). β-adrenergic signaling has been linked to inactivation of dendritic Kv1.1 resulting in increased dendritic excitability (Liu et al., 2017). Moreover, noradrenergic modulation was implicated in tuning spike timing dependent plasticity in the visual cortex through activation of AC and PLC cascades (Seol et al., 2007; Huang et al., 2012).

5-HT is synthesized by neurons in the Raphe nuclei enabling serotonergic signaling involved in various physiological processes, including sleep, social behavior, sexual activity, learning and memory, pain, and feeding (Bockaert et al., 2006). Serotonergic inputs into neural networks operate via at least 15 structurally and pharmacologically different receptors (Hoyer et al., 1994) and trigger various responses. Among them are hyperpolarization-induced decrease in neural firing rate, stimulation of PLC via G_q_ proteins, initiating intracellular Ca^2+^ and diacylglycerol signaling, and activation or inactivation (G_s_ or G_i/o_) of AC to regulate cyclic AMP (cAMP) levels (Filip and Bader, 2009). Within the dorsal cochlear nucleus (DCN), serotonin enhanced the excitability of fusiform principal cells through the activation of 5-HT receptors 5-HT_2A_/_2C_R and 5-HT_7_R via HCN current augmentation (Tang and Trussell, 2015). Furthermore, 5-HT_7_R activation upregulated the phosphorylation of synapsin-1 and the consequent decrease SV replenishment in stem-cell-derived human neurons (Patzke et al., 2019). In addition, the 5-HT_1B_R, expressed presynaptically, reduced GABA_A_ signaling in the inferior colliculus in the auditory midbrain and thus facilitated higher spiking rates of inferior colliculus neurons (Hurley et al., 2008). Serotonin facilitated long-term depression of glutamatergic transmission at the medium spiny neurons in the nucleus accumbens by activating 5-HT_1B_Rs (Huang et al., 2013). In the hippocampal dentate gyrus of rats, the 5-HT_4_R had an inhibitory effect on long-term potentiation (Kulla and Manahan-Vaughan, 2002).

The presence of noradrenergic and serotonergic innervation of the CN of rat and cat has been indicated through biochemical and fluorescence-based assays (Kromer and Moore, 1976; Klepper and Herbert, 1991; Cransac et al., 1995; Thompson and Thompson, 2001). However, a detailed analysis of neuromodulation in the CN remained to be performed. Here, we used SBEM of the CN and found varicose neurites indicative of monoaminergic innervation. We then focused on studying neuromodulation of the mouse endbulb synapse of SGNs and bushy cells in the AVCN by NE and 5-HT. Immunohistochemistry revealed the presence of the norepinephrine and serotonin transporters NET and SERT near the endbulb synapse as well as the expression of α_2C_-AR, 5-HT_1B_R, 5-HT_4_R, and 5-HT_7_R receptors in/near BCs supporting the hypothesis of serotonergic and adrenergic modulation in the AVCN. Finally, we performed whole-cell patch-clamp recordings from BC to evaluate the effects of administered NE and 5-HT on the spontaneous and evoked synaptic transmission as well as on the electrical properties of BCs.

## 2 Materials and methods

### 2.1 Animals

Mice from the wild-type substrain C57BL/6N were used for the electrophysiology and immunostaining experiments. They were obtained from the colony maintained at Max Planck Institute for Multidisciplinary Sciences, Faßberg Campus, Göttingen. Male and female mice, of ages ranging between P14 and P20, were sacrificed by decapitation for then dissecting the brain and preparing acute brainstem slices. Mice aged p15 to p22, as well as a p42 mouse, were used in our immunohistochemical assays. The use of animals complied with national animal care guidelines in the registered facility 33.23-42508-066-§11. For the electron microscopy experiments, an 8-week-old C57BL/6J mouse was used to make VCN sample and two 7-week-old CBA/Ca mice were used to make DCN samples. The C57BL/6J mouse was purchased from Shanghai Jihui Laboratory Animal Care Co., Ltd., and the two CBA/Ca mice were purchased from Sino-British: SIPPR/BK, Lab. Animal Ltd (Shanghai, China). The experiments complied with national animal care guidelines and were approved by the Institutional Authority for Laboratory Animal Care of Shanghai Ninth People's Hospital (SfH9H-2020-A65-1).

### 2.2 SBEM sample preparation

The mice were anesthetized with 2% isoflurane inhalation before successive transcardial perfusions of 15 ml sodium cacodylate buffer (0.15 M) and 30 ml mixed fixative solution containing 2% paraformaldehyde and 2.5% glutaraldehyde (buffered by 0.08 M sodium cacodylate, pH = 7.4). After decapitation, the brains were exposed by removing the skull and post-fixed by immersion in the same fixative at 4°C for 24 h. Then, the specimen was transferred to sodium cacodylate buffer (0.15 M) in a Petri dish (on ice), and the brainstem was exposed by carefully removing the cerebellum under a dissecting microscope. The CN samples were harvested from the brainstem by cutting with a scalpel blade.

The en bloc staining for SBEM was performed following the previously published protocol (Hua et al., 2015, 2022) with minor modifications. In brief, the CN samples were washed twice in 0.15 M cacodylate (pH 7.4) for 30 min each and sequentially immersed in 2% OsO_4_ (Ted Pella), 2.5% ferrocyanide (Sigma), and again 2% OsO_4_ at room temperature (RT) for 2, 1.5, and 1 h, respectively, without intermediate washing step. All staining solutions were buffered with 0.15 M cacodylate buffer (pH 7.4). After being washed sequentially in 0.15 M cacodylate buffer and nanopore-filtered water for 30 min each, the samples were incubated at RT in 1% thiocarbohydrazide (aqueous solution) for 1 h and further stained with 2% OsO_4_ aqueous solution for 2 h, 1% uranium acetate at 4°C for 8 h and at 50°C for 2 h, as well as 0.03 M lead aspartate solution (pH 5.0 adjusted by KOH, Electron Microscopy Sciences) at 50°C for 2 h. Between steps, double rinses in nanopore-filtered water for 30 min each were performed.

For resin embedding, the samples were dehydrated through a graded acetone series (50%, 75%, and 90%, for 30 min each at 4°C) into pure acetone (3 x 100%, 45 min at RT), followed by infiltration with 1:1 mixtures of acetone and Spurr's resin monomer (4.1 g ERL 4221, 0.95 g D.E.R^TM^ 736, 5.9 g NSA, and 1% 2-dimethylaminoethanol, DMAE; Sigma-Aldrich) at RT for 8 h on a rotator. Infiltrated samples were then incubated in pure resin for 8–12 h before being placed in embedding molds (Polyscience, Germany) and incubated in a pre-warmed oven (70°C) for 72 h.

### 2.3 SBEM imaging

The embedded samples of AVCN and DCN were trimmed to a block face of 600 × 600 μm and coated with thin-layer gold (thickness of 30 nm) by a high-vacuum coating device (ACE600, Leica, Germany). It was imaged using a field-emission SEM (Gemini300, Zeiss) equipped with an in-chamber ultramicrotome (3ViewXP, Gatan). Focal charge compensation was set to 100% with a high vacuum chamber pressure of 2.8 × 103 mbar. Serial images were acquired in a stitching mode at 12 nm or 15 nm pixel size, nominal cutting thickness of 35 or 50 nm, incident beam energy of 2 keV, and dwell time of 1.5 μs.

### 2.4 Electrophysiology solutions

Dissections were performed in an ice-cold cutting solution containing (in mM): 50 NaCl, 26 NaHCO_3_, 1.25 NaH_2_PO_4_H_2_O, 2.5 KCl, 20 glucose, 0.2 CaCl_2_, 6 mM MgCl_2_, 0.7 Na L-ascorbate, 2 Na pyruvate, 3 myo-inositol, 3 Na L-lactate, and 120 sucrose adjusted to pH 7.3–7.4. Recordings were performed in solutions based on artificial cerebrospinal fluid (aCSF), containing (in mM): 125 NaCl, 26 NaHCO_3_, 1.25 NaH_2_PO_4_H_2_O, 2.5 KCl, 13 glucose, 2 CaCl_2_, 1 MgCl_2_, 0.7 Na L-ascorbate, 2 Na pyruvate, 3 myo-inositol, and 3 Na L-lactate adjusted to pH 7.3–7.4. aCSF was supplemented with 10 μM bicuculline, a competitive antagonist of GABA_A_ receptors and 2 μM strychnine to inhibit postsynaptic glycine receptors (control solution). The test solution additionally contained either 100 μM NE or 10 μM 5-HT. All solutions were continuously aerated with carbogen (95% O_2_, 5%CO_2_).

For the HCN and ${\text{K}}_{\text{LVA}}^{+}$ experiments, the control and test solutions additionally contained 1 μM tetrodotoxin (TTX, Alomone Labs) blocking voltage-sensitive sodium currents, 0.25 mM CdCl_2_ blocking the voltage-sensitive Ca^2+^ currents, and 10 μM 6,7-dinitroquinoxaline-2,3-dione (DNQX) to block excitatory postsynaptic currents diluted in dimethyl sulfoxide (DMSO). To measure HCN currents, additional 25 nM α-dendrotoxin (α-DTX, Alomone labs) was added to block ${\text{K}}_{\text{LVA}}^{+}$. For the measurement of ${\text{K}}_{\text{LVA}}^{+}$ currents, we included 10 μM ZD7288 (diluted in DMSO) to block HCN channels. The final concentration of DMSO in our bathing solution for both experiments was 0.1%.

During the fiber stimulation experiments, the control solution was supplemented with 1 mM kynurenic acid sodium salt (Abcam Biochemicals, Cambridge, UK), a low-affinity AMPAR antagonist, to prevent receptor saturation/desensitization.

The pipette solution contained (in mM): 115 K-gluconate, 8 EGTA, 10 HEPES, 4 Mg-ATP, 0.3 Na-GTP, 10 Na_2_Phosphocreatine, 4,5 MgCl_2_, 10 NaCl, pH 7.3, and 317 mOsm. In addition, the fluorescent dye Alexa 568 (34 μM, Invitrogen) was added to the intracellular solution to assist identification of the cell type which we observed using a HXP 120184 mercury lamp, with a FITC filter set (Semrock). For the fiber stimulation experiments, we added 1 N-(2,6-dimethylphenyl carbamoylmethyl) triethylammonium chloride (QX-314; Alomone Labs, Jerusalem, Israel) to the intracellular solution to block sodium channels. The source of the above listed chemicals was Merck, Germany, unless stated differently.

### 2.5 Slice preparation for electrophysiology

Following sacrifice of mice, their brains were immediately immersed in ice-cold cutting solution. A midsagittal cut was performed on the brain, and the hindbrain was severed from the forebrain. This was followed by gluing the hindbrain to the stage of the vibratome (VT 1200S, Leica, Wetzlar, Germany). To obtain parasagittal brainstem slices, a 0.02 mm/s advancing rate of the cutting blade and vibration amplitude of 1.5 mm were used. Then, 150 μm thick parasagittal slices of the CN were cut and incubated for 30 min in aCSF at 35°C. The dissection materials were as follows: forceps, small surgical scissors, surgical scissors, wax stage, dissection blade, glass dropper (all Fine Science Tools, Heidelberg, Germany), cyanoacrylate glue Loctite 401 (Henkel, Germany).

### 2.6 Electrophysiological recordings

Patch pipettes were made from borosilicate glass (Science products, GB150F-8P) and pulled using a P-97 Flaming/Brown micropipette puller (Sutter Instruments). The pipettes had resistances between 2.5 and 4.5 MΩ. The slices were placed in a custom-made stage on an Axioscope 2 FS plus microscope (Zeiss, Jena, Germany) and held in place with a custom-made stainless-steel harp with glued nylon strings. The slices were visualized with a 40× water-immersion objective, using differential interference contrast illumination. Patch-clamp recordings were made from BC of the AVCN using an EPC10 USB double patch-clamp amplifier, which was controlled by the PatchMaster software (HEKA Elektronik). The sampling interval was 25 μs, and data were filtered at 7.3 kHz. The liquid junction potential of +12 mV was corrected through the PatchMaster software, and cells were voltage-clamped at a holding potential of −70 mV. Mean series resistance was ~5 MΩ and was compensated up to 70% with a 10 μs lag. Recordings, which displayed leak currents beyond −150 pA at −70 mV or a series resistance (R_s_) above 10 MOhm, were discarded. All experiments were performed at near physiological temperature (32–35°C) maintained by constant superfusion (flow rate 3–4 ml/min) of aCSF, heated by an inline solution heater (SH-27B174 with TC-324B controller; Warner Instruments, Hamden, CT, USA) and monitored by a thermistor placed between the inflow site and the slice, in the recording chamber.

The recordings of mEPSCs, current threshold, and rate of depolarization from cells that compose our control dataset speed were made in the following fashion: after achieving whole-cell configuration while perfusing aCSF supplemented with 10 μM strychnine, we tested for the cell identity. This was done by briefly recording mEPSCs [BC mEPSCs show shorter decay times then stellate cells (Lu et al., 2007)] as well as current clamp protocols testing the sensitivity to speed of depolarization, the firing patterns of the cells evoked by stepwise current injection (BCs show phasic, stellate cells tonic firing). For estimating the rate of depolarization, we documented the slowest rate at which an AP is generated and analyzed the first derivative of the voltage trace before the generation of the AP. To estimate the current threshold, we injected currents with different amplitudes and duration and documented the first current value at which an action potential (AP) was generated. Once we had determined the cell to be a BC, we perfused the control solution for 1 min. At 1 min of control solution perfusion, we recorded mEPSC for 1 min, right after that we performed the above-mentioned current clamp protocols. Each of these recordings was performed once per cell. The recordings from cells exposed to 5-HT or NE were performed in a similar fashion, where, after we determined the cell type to be BC, we perfused the test solution containing 5-HT or NE. At 5 min of perfusion, we recorded mEPSCs, followed by the aforementioned current clamp protocols which were applied once in the recording.

The ${\text{K}}_{\text{LVA}}^{+}$ and HCN channel experiments were performed separately for control, 5-HT, and NE conditions after incubation with the respective solution for 5 min. For both types of experiments, recordings were made at 30 s, 2 min, and 5 min of NE perfusion. Due to the lack of statistically significant difference, we only described the recordings at 5 min of perfusion. In the HCN channel experiments, we voltage-clamped the cell and changed the voltage in steps of 5 mV from −57 to −112 mV. For the ${\text{K}}_{\text{LVA}}^{+}$ experiments, we used voltage steps of 5 mV from −90 to −40 mV. We derived the HCN currents at the plateau at −112 mV and the peak ${\text{K}}_{\text{LVA}}^{+}$ currents at −40 mV and then normalized them by the capacitance of the cell to derive the current density. The cell capacitance was recorded as the C-slow value at the timepoint of establishing whole cell configuration. As a negative control for the HCN experiments, 10 μM of ZD7288 was added to this solution to block HCN currents. As a negative control for the ${\text{K}}_{\text{LVA}}^{+}$, 50 nM α-DTX was added to block ${\text{K}}_{\text{LVA}}^{+}$ (DTX was diluted in PBS, no further additives).

For studying effects on evoked endbulb transmission, we minimally stimulated presynaptic auditory nerve fibers forming the endbulbs of Held with a monopolar electrode in a patch pipette filled with aCSF, placed at a distance of at least three cell diameters from the cell being recorded. Stimulating currents of 7–24 μA were delivered through a stimulus isolator (A360 World Precision Instruments, Sarasota, FL, USA). We performed recordings of regular trains of 35 evoked EPSC (eEPSCs) at 100 Hz and 200 Hz. The inter stimulus interval between trains was 40 s to allow for the refilling of the high Pvr pool and preventing short-term plasticity effects from influencing subsequent trials. We perfused the control solution for this experiment for 2 min before recording. Next, we perfused the NE or 5-HT containing solutions for 5 min before starting to record the second set of data.

### 2.7 Sample preparation for immunohistochemistry

The whole brain was submerged in 4% formaldehyde (FA, Carl Roth, Karlsruhe, Germany) in PBS (Merck, Germany) for fixation, right after detachment from the skull. The brain was fixed for 24 h at 4°C, and, thereafter, the solution was changed to 30% PBS-based sucrose solution to dehydrate the sample and prepare it for the next step of cryosectioning. The brains were incubated in the sucrose solution for ~2 days until they sunk to the bottom of the falcon tube. The cryostat was set to −22°C for the chamber and to −23°C for the stage. Before sectioning, the forebrain and hindbrain were separated, and the dehydrated hindbrains were attached to the cutting stage by immersion in the Cryomatrix (Thermo Fisher Scientific, Waltham, MA, USA). This was followed by coronal cryosectioning in a caudal to rostral direction. The anatomical landmark for reaching the level of the AVCN is the 7th cranial nerve which appears as an opaque white line traversing the brainstem in a ventrolateral to dorsomedial orientation. Subsequent 30 μm thick cryosections, containing AVCN, were collected on electrostatically positively charged microscope slides (Thermo Fisher Scientific, MA, USA) to a total amount of eight slices of each ACVN. The collected slices were kept at −20°C before fixation and staining. Before staining, each slice was incubated with blocking solution (goat serum dilution buffer, GSDB: 16% normal goat serum, 450 mM NaCl, 0.6% Triton X-100, 20 mM phosphate buffer) for 1 h.

### 2.8 Staining and microscopy

All antibodies (Ab) were diluted in GSDB. For staining of monoamine transporters, antibodies against norepinephrine (NET), and serotonin (SERT) transporters were diluted 1:200 and for counterstaining the presynaptic marker vGlut1 guinea-pig-anti-vGlut1 was diluted 1:2,000. In the case of the monoamine receptor staining, primary antibody combinations routinely contained guinea-pig-anti-vGlut1 Ab (1:1,000 or 1:2,000, Synaptic Systems GmbH, Göttingen, Germany) and chicken anti-Homer1 Ab (1:500, Synaptic Systems GmbH, Göttingen, Germany), labeling the excitatory postsynaptic density inside of the principal cell. To label for the various receptors, a relevant rabbit Ab (1:200 or 1:500) was included. In parallel, a negative control staining with the same components and with additional blocking peptide for each of the tested receptors was performed. The concentration of the blocking peptide was 500 higher than that of the antibody in the final solution. Before applying to the tissue, both solutions were kept at room temperature and centrifuged at 2,000*g* for an hour. All the slices were incubated with the primary antibodies overnight at 4°C.

This was followed by washing 3 × 10 min first with washing buffer (20 mM phosphate buffer, 0.3% Triton X-100, 0.45 M NaCl) and then 3 × 10 min with PBS. The slides were dried off of PBS, and secondary antibodies goat-anti-rabbit Alexa fluor 488, goat-anti-chicken/mouse Alexa fluor 568, and guinea-pig-anti-mouse Alexa fluor 633/647 were added at a dilution of 1:200. The incubation with secondary Abs lasted 2 h and was followed by washing 3 × 10 min with wash buffer and 3 × 10 min with PBS. Next, the slides were washed in 5 mM phosphate buffer for 5 min and mounted with 50 μl of fluorescence mounting medium based on Mowiol 4–88 (Carl Roth, Karlsruhe, Germany) and covered with a thin glass coverslip. The slides were kept at 4°C until imaging. The list of all antibodies used can be found in Tables 1, 2.

**Table 1:** Primary antibodies used in the immunolabeling experiments.

**Primary antibodies**	**Source**
Mouse-anti-Norepinephrine transporter	Synaptic Systems GmbH, Göttingen, Germany
Rabbit-anti-Serotonin transporter	Synaptic Systems GmbH, Göttingen, Germany
Guinea-pig-anti-vGlut1	Synaptic Systems GmbH, Göttingen, Germany
Chicken-anti-Homer1	Synaptic Systems GmbH, Göttingen, Germany
Mouse-anti-Gephyrin	Synaptic Systems GmbH, Göttingen, Germany
Rabbit-anti-5-HT_1A_ receptor + blocking peptide	Alomone Labs, Israel
Rabbit-anti-5-HT_1B_ receptor + blocking peptide	Alomone Labs, Israel
Rabbit-anti-5-HT_2A_ receptor + blocking peptide	Alomone Labs, Israel
Rabbit-anti-5-HT_2B_ receptor + blocking peptide	Alomone Labs, Israel
Rabbit-anti-5-HT_4_ receptor + blocking peptide	Alomone Labs, Israel
Rabbit-anti-5-HT_5B_ receptor + blocking peptide	Alomone Labs, Israel
Rabbit-anti-5-HT_7_ receptor, + blocking peptide	Alomone Labs, Israel
Rabbit-anti-α_1B_-AR, + blocking peptide	Alomone Labs, Israel
Rabbit-anti-α_1D_-AR, + blocking peptide	Alomone Labs, Israel
Rabbit-anti-α_2C_-AR, + blocking peptide	Alomone Labs, Israel
Rabbit-anti-β_1_-AR, + blocking peptide	Alomone Labs, Israel
Rabbit-anti-β_2_-AR, + blocking peptide	Alomone Labs, Israel
Rabbit-anti-β_3_-AR, + blocking peptide	Alomone Labs, Israel

**Table 2:** Secondary antibodies used in the immunolabeling experiments.

**Secondary antibodies**		**Source**
**Fluorophore**	**Coupled to, Ab**	
Alexa fluor 488	Goat-anti-guinea-pig	MoBiTec (Invitrogen), Göttingen, Germany
Alexa fluor 488	Goat-anti-rabbit	MoBiTec (Invitrogen), Göttingen, Germany
Alexa fluor 568	Goat-anti-guinea-pig	MoBiTec (Invitrogen), Göttingen, Germany
Alexa fluor 568	Goat-anti-rabbit	Thermo Fisher, Waltham, Massachusetts, US
Alexa fluor 568	Goat-anti-chicken	Abcam, Cambridge, UK
Alexa fluor 633	Goat-anti-guinea-pig	Thermo Fisher, Waltham, Massachusetts, US
Alexa fluor 647	Goat-anti-guinea-pig	Thermo Fisher, Waltham, Massachusetts, US
Alexa fluor 647	Goat-anti-rabbit	MoBiTec (Invitrogen), Göttingen, Germany
Alexa fluor 633	Goat-anti-mouse	Thermo Fisher, Waltham, Massachusetts, US
Abberior Star 635p	goat-anti-mouse 635p	Abberior, Göttingen, Germany
Abberior Star 580	Goat-anti-guinea-pig	Abberior, Göttingen, Germany

Two-color STED images of cryosections stained for monoamine transporters were acquired using a STEDYCON (Abberior Instruments GmbH, Göttingen, Germany) with a 100× oil immersion objective. Images of the monoamine receptor preparations were taken with Zeiss confocal laser scanning microscopes 780 and 880 with 40× or 100× oil immersion objectives.

### 2.9 Data analysis

#### 2.9.1 SBEM image processing, neurite reconstruction, and quantification

The dataset was aligned using a self-written MATLAB script based on cross-correlation maximum between consecutive slices. Then, the aligned datasets were split into cubes (1,024 × 1,024 × 1,024 voxels) for viewing and tracing in a browser-based annotation tool, webKNOSSOS as previously described (Gour et al., 2021; Hua et al., 2022; Jiang et al., 2025). Neurites with associated mitochondria, dense core vesicles and synaptic vesicle clouds, were volume traced by human annotators.

#### 2.9.2 Patch-clamp analysis

Data were analyzed with Igor pro 6.3 software (Wavemetrics) using custom written programs. Graphs were made with custom written programs in Igor pro 6.3 or GraphPad Prism 9 for Windows, GraphPad Software, Boston, Massachusetts USA, www.graphpad.com and assembled with Inkscape (Inkscape Project, 2020). mEPSCs were detected and analyzed with the NeuroMatic toolkit in Igor (Rothman and Silver, 2018). The average mEPSCs' amplitude from our control and NE dataset were used to determine the quantal size of synaptic release. The values were normalized for the amplitude reduction after perfusion of kynurenic acid by the empirically derived factor of 3.23.

#### 2.9.3 Analysis of confocal and STED data

The obtained images were processed with Fiji (Schindelin et al., 2012). The Z stacks obtained from the monoamine receptor staining were transformed into maximum intensity projections. Following that, the immunolabeling figures were created with Inkscape (Inkscape Project, 2020).

#### 2.9.4 Statistical analysis

For the fiber stimulation experiments, we used consecutive recordings from the same cells: first subjecting them to a control solution, followed by the test recording in 5-HT or NE solution. For all other experiments, we pooled the data in the different datasets from different cells. The data were analyzed using Excel and Igor Pro software. The data are presented as mean ± standard error of the mean (SEM). The number of the animals is indicated as n and the number of cells as N. For two sample comparisons, normality of the distributions (Jarque-Bera test) and equality of variances (*F*-test) were tested. This was followed by Student's *t*-test or Mann–Whitney–Wilcoxon test in case the normality and/or equality of variances criteria were not met. Significant differences are presented as ^*^p < 0.05, while non-significance is presented as n.s. *p* > 0.05.

## 3 Results

### 3.1 Electron microscopical correlates of monoaminergic innervation of the cochlear nucleus

**Figure 1:**
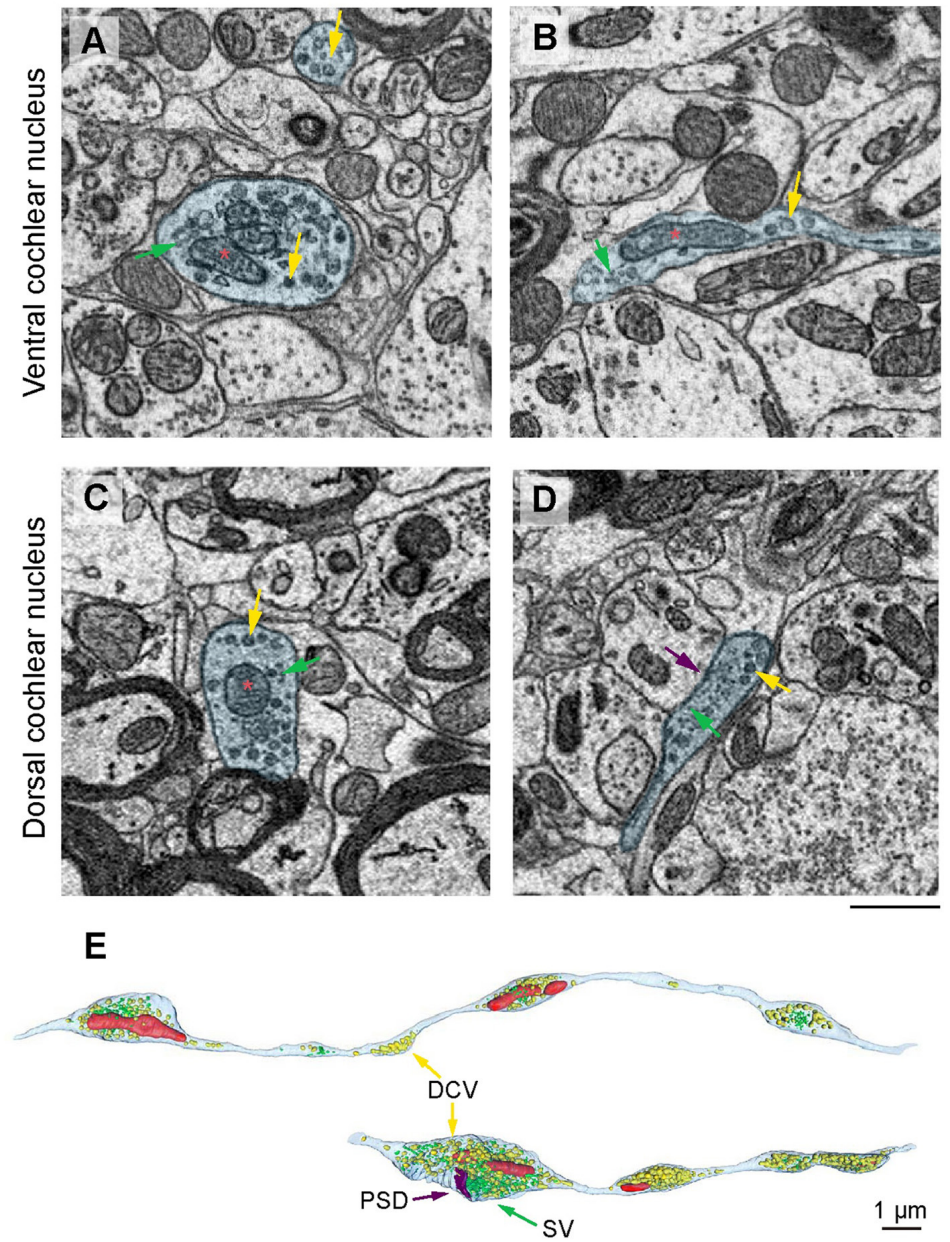
Electron microscopical evidence for putative monoaminergic varicosities in the CN. **(A–D)** Instances of varicosities (blue) filled with mitochondria (red asterisks), a mixture of synaptic vesicles (SVs, green arrows), and dense core vesicles (DCVs, yellow arrows). Scale bar, 500 nm. **(E)** 3D reconstruction of the varicosity-forming projections (outlined with cyan) filled with DCV (yellow arrows) and synaptic vesicles (green), mitochondria (red), and putative postsynaptic density (PSD, purple). Scale bar, 1 μm.

Noradrenergic varicosity diameters in the mouse olfactory bulb were reported to be ~0.2 μm in transverse diameter (Horie et al., 2021), while in rat neocortex in the range of 0.4 μm to 1.2 μm (Séguéla et al., 1990). Dopaminergic varicosities were reported to be of an average transverse diameter of 0.24 μm (Descarries et al., 1996). Dense core vesicles were observed in 5-HT and NE neurons in addition to clear core SVs (Suzuki et al., 2015; Horie et al., 2021). Hence, we performed scanning electron microscopy on large sample block faces of the en bloc-stained CN tissues of mice (Jiang et al., 2025). This allowed us to search for varicosities containing dense core vesicles within both AVCN (Figures 1A, B) and DCN (Figures 1C, D) subdivisions. Next, we acquired two small EM volumes using SBEM to 3D reconstruct the candidate projections with consecutive varicosities, which contained dense core vesicles (DCVs). The swellings contained synaptic vesicles, DCV, and sometimes mitochondria and were approximately 1 μM in transverse diameter (Figure 1E). In one of our samples (Figure 1E), a putative postsynaptic density was found. Hence, our SBEM data point out the presence of varicosity-like neurites in the CN and future investigations on larger EM volumes of AVCN will be needed to comprehensively characterize this type of neurite.

### 3.2 Immunohistochemical verification of monoaminergic innervation in the cochlear nucleus

**Figure 2:**
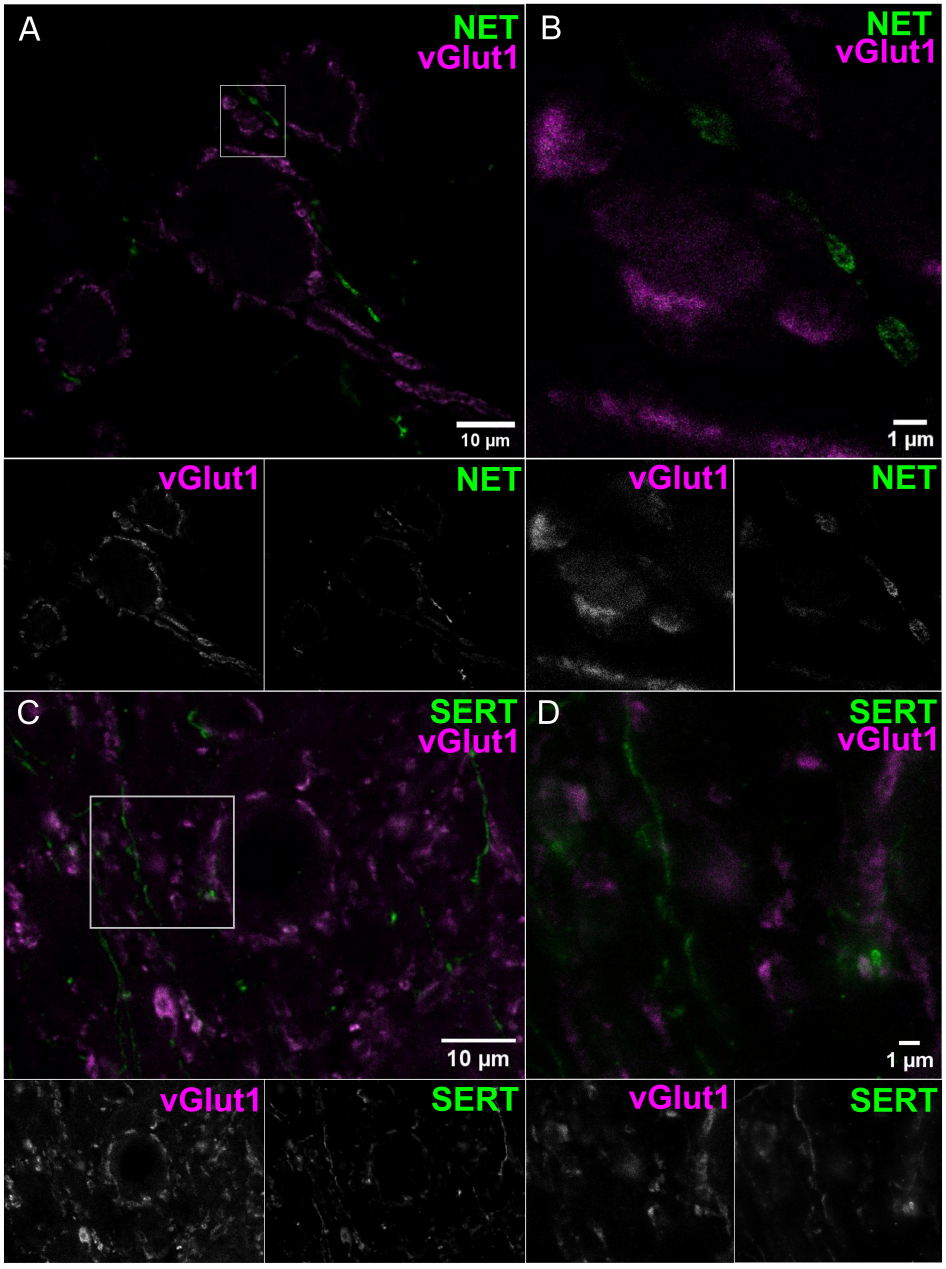
Noradrenergic and serotonergic innervation located near endbulbs of Held. Coronal sections of AVCN were used for confocal **(A, C)** and STED **(B, D)** imaging. **(A, B)** Endbulbs labeled by vGlut1 (magenta) and NET-positive structures (green), similar results from three animals. **(C, D)** Endbulbs labeled by vGlut1 (magenta) and SERT neurites (green) neighboring the endbulbs, similar results from three animals. Both transporter Ab display grainy signal forming strings, with transverse diameter <1 μm. Scale bars 1 μm.

To obtain molecular evidence for monoaminergic innervation of the AVCN, we probed for the presence of the NE and 5-HT transporters, NET and SERT, respectively, using confocal imaging and STED nanoscopy. Endbulbs were labeled by staining for the vesicular glutamate transporter 1 (vGlut1, 1:2,000 dilution). We observed NET positive varicosities apposed around endbulbs (Figure 2A confocal overview and Figure 2B STED image, 1:200 dilution, representative for *n* = 3 animals). We observed the SERT (Figure 2C confocal overview and Figure 2D STED image, 1:200 dilution, representative for *n* = 3 animals) in the vicinity of the endbulbs. In both cases, we observed a grainy signal forming string-like structures, smaller than 1 μm in transverse diameter, in agreement with the electron microscopical observations reported above. The SERT signal appeared smaller in diameter. To verify the NET and SERT signal, we labeled slices of the locus coeruleus and the medial raphe nuclei, respectively, as positive controls (Supplementary Figure S1).

Next, we probed for the presence of monoamine receptors in endbulbs and BCs, using vGlut1 as a presynaptic marker and Homer1 as excitatory postsynaptic marker. We shortlisted 5-HT and NE receptors according to three transcriptomic databases for SGNs (Shrestha et al., 2018; Sun et al., 2018; Li et al., 2020). Initially, we probed for β_1_-AR, β_2_-AR, β_3_-AR, α_1B_-AR, 5-HT_1A_R, 5-HT_2A_R, 5-HT_2B_R, and HT_5B_. In each case, we optimized the staining protocol to improve labeling (Supplementary Methods, Supplementary Figure S2). Despite the optimization, we could not detect convincing immunofluorescence except for 5-HT_5B_R (Supplementary Figure S2). In some cases, we experienced bleed-through (examples in Supplementary Figure S2). Next, we probed for the receptors 5-HT_7_R, 5-HT_2B_R, α_1B_-AR, α_1D_-AR, and α_2C_-AR, again involving optimization, with a protocol that stained cryosections of immersion-fixed brainstems with the antibody against the receptor of interest (1:500) and vGlut1 (1:2,000) as a presynaptic marker and Homer1 (1:500) as postsynaptic marker (Supplementary Figure S2). As a result, we observed a convincing staining for 5-HT_7_R and α_2C_-AR.

**Figure 3:**
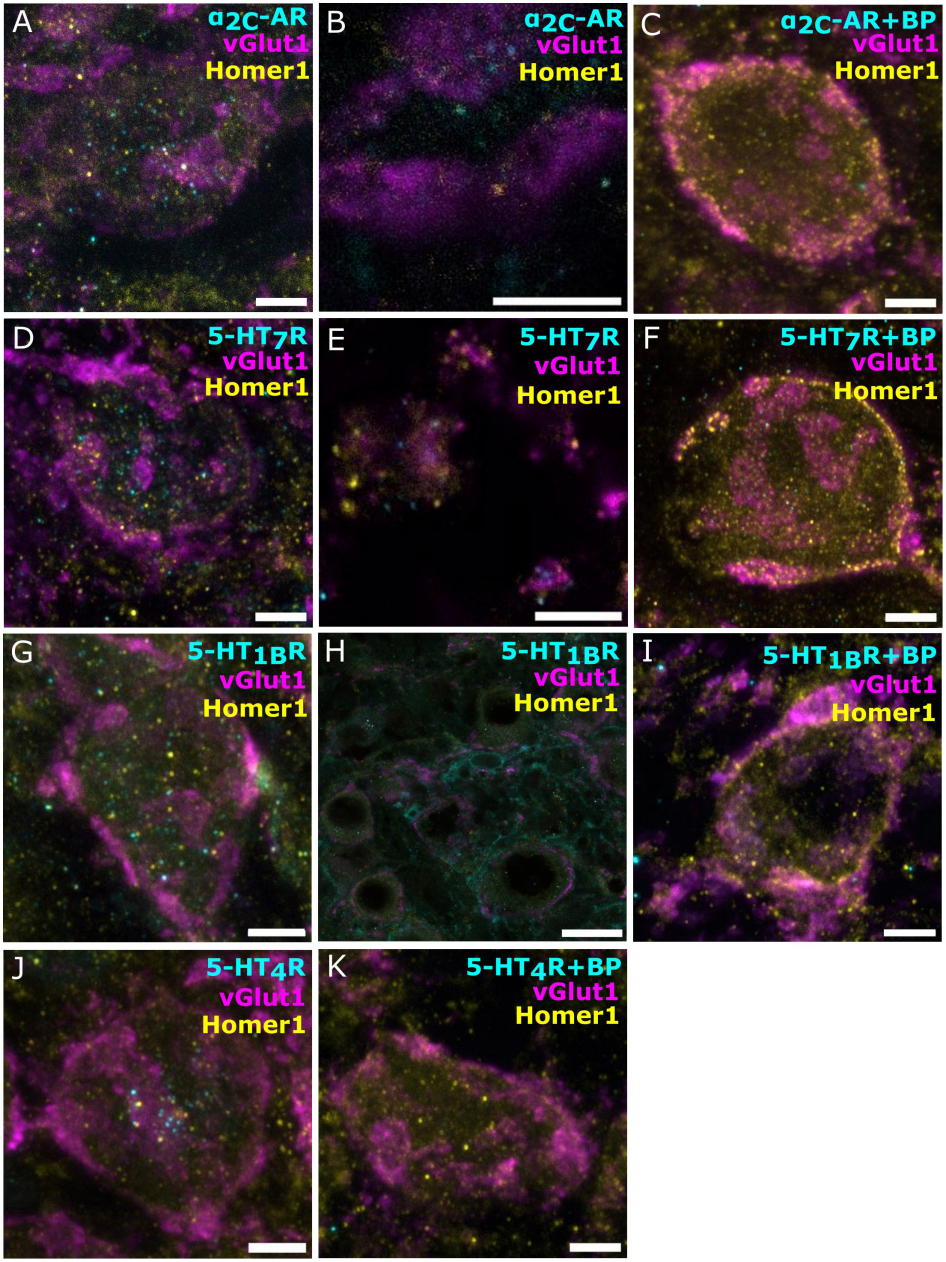
Immunofluorescence for norepinephrine and serotonin receptors in bushy cells and near endbulbs. Confocal sections of the AVCN with vGlut1 marking the presynaptic endbulbs of Held and Homer1 as postsynaptic marker. **(A–C)** α_2C_-AR (cyan, Alexa-fluor-488), vGlut1 (magenta, Alexa-fluor-647/633), and Homer1 (yellow, Alexa-fluor-568). **(A)** Grainy α_2C_-AR signal, similar results from four animals. **(B)** A Zoom-in of the top view of a BC where α_2C_-AR (cyan) is located closely to the endbulbs (magenta). **(C, A)** Blocking peptide for the Ab against α_2C_-AR was added as a negative control. **(D–F)** 5-HT_7_R (cyan, Alexa-fluor-488), markers vGlut1 (magenta, Alexa-fluor-633), and Homer1 (yellow, Alexa-fluor-568). **(D)** Grainy 5-HT_7_R signal, similar results from four animals. **(E)** Zoom-ins of the top view of BC where 5-HT_7_R signal (cyan) is located closely to the endbulbs (magenta), **(F**, **A)** negative control, a blocking peptide for the Ab against HT_7_R was. **(G–I)** 5-HT_1B_ receptor (cyan, Alexa-fluor-488), markers vGlut1 (magenta, Alexa-fluor-647), and Homer1 (yellow, Alexa-fluor-568). **(G)** Grainy 5-HT_4_R signal, similar results from three animals. **(H)** The 5-HT_1B_R signal is also found in the vicinity of the BCs on smaller structures. **(I**, **A)** blocking peptide for the Ab against 5-HT_1B_R was added as a negative control. **(J**, **K)** 5-HT_4_ receptor (cyan, Alexa-fluor-488), markers vGlut1 (magenta, Alexa-fluor-647), and Homer1 (yellow, Alexa-fluor-568). **(J)** Grainy 5-HT_4_R signal, similar results from three animals. **(K**, **A)** blocking peptide for the Ab against 5-HT_4_R was added as a negative control. Scale bar 5 μm for all except **(H)**. **(H)** Scale bar 20 μM.

The α_2C_-AR immunofluorescence was mainly situated within the BC soma and their plasma membrane, possibly indicating cytoplasmic reserves or intermediate stages of receptor turnover (Figure 3A). Less frequently, the signal was located in the vicinity of the endbulb (Figure 3B, a close-up on the endbulbs from a top view of the BCs). Notably, the negative control (Figure 3C) suggested specificity of the immunolabeling. Similarly, the majority of the specific 5-HT_7_R immunofluorescence was located within the BC soma and in their plasma membrane (Figure 3D). Occasionally, we found 5-HT_7_R signal close to the endbulbs (Figure 3E). Furthermore, signal was also detected in the vicinity of the BC, possibly reflecting labeling of other neuronal structures, such as potential projections from stellate cells. The negative control (Figure 3F) indicates specificity of the 5-HT_7_ receptor immunolabeling on Figure 3D. Similarly, we found the receptors 5-HT_1B_ and 5-HT_4_ expressed within the BC soma (Figures 3G, J, respectively). The staining against the 5-HT_1B_R also showed signal, outlining smaller structures in proximity to, yet distinct from BCs (Figure 3H). The 5-HT_4_R signal appeared mainly cytoplasmic. We performed negative controls, confirming the specificity of the signal (Figures 3I, K, respectively). In summary, we provided positive monoamine transporter and receptor staining, suggesting noradrenergic and serotonergic innervation of the AVCN.

### 3.3 Studying the effects of NE or 5-HT on neurotransmission at the endbulb synapse

**Figure 4:**
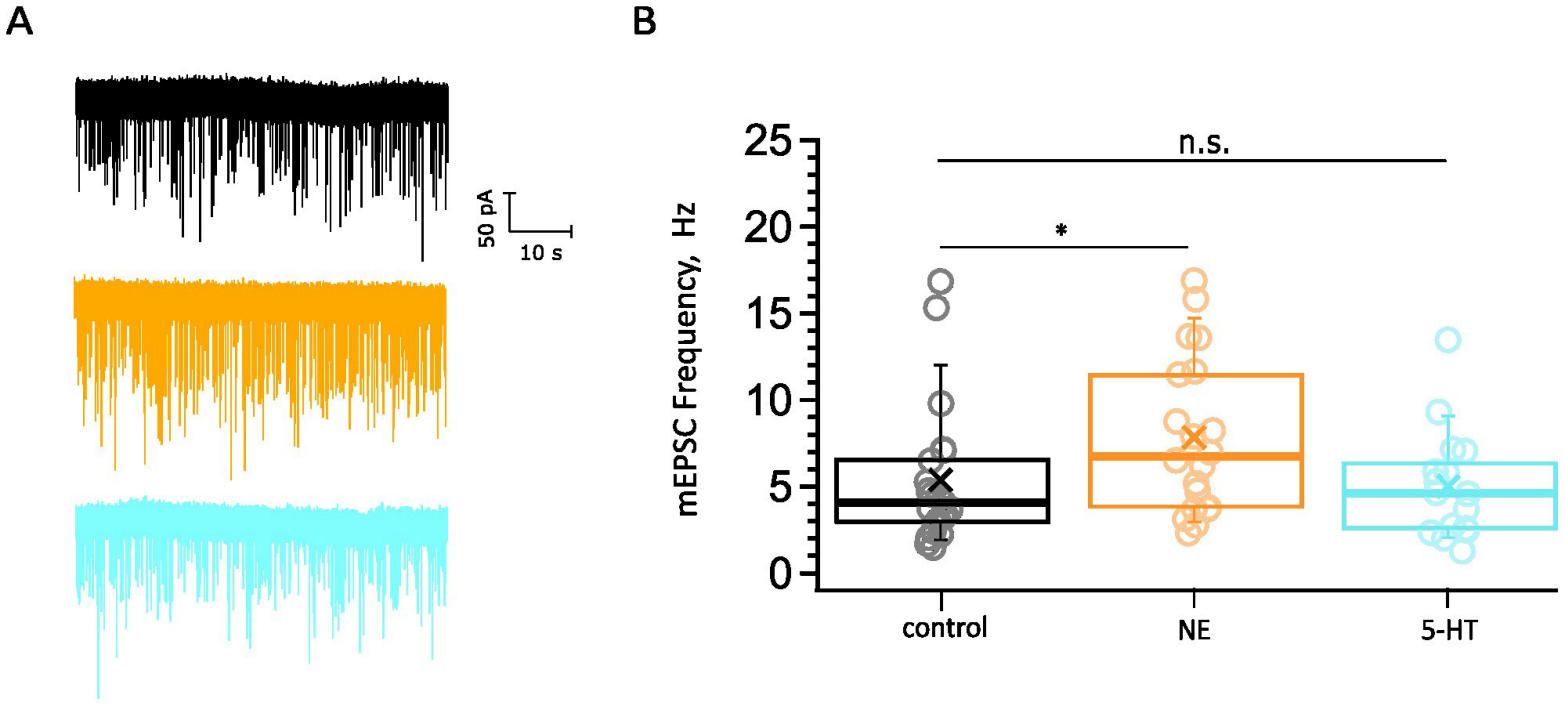
Probing for potential neuromodulation of spontaneous synaptic transmission. **(A)** Examples of mEPSC recordings: control (black), NE (orange), 5-HT (cyan). Scale bar 50 pA, 10 s. The datasets consist of pooled recordings comparing mEPSCs of BC in a control bathing solution with mEPSCs of BC in 100 μM NE (orange) or 10 μM 5-HT (cyan) containing solution. **(B)** The mEPSC frequency is increased upon the application of NE (8,538 mEPSCs, *n* = 20 cells from 9 mice, *p* = 0.049) compared to control recordings (5.37 ± 0.88 Hz, 5,414 mEPSCs, *n* = 21 cells from 14 mice). The mEPSC frequency remained unaltered upon the application of 5-HT (4,821 mEPSCs, *n* = 16 cells from 7 mice, *p* = 0.95). The data are represented as box plots with minimum, first quartile, median, third quartile, maximum, and a cross representing the mean. Each data point represents an average of the given parameter across the events recorded from one cell exposed to one condition. The ranges of mEPSCs recorded for 1 min were as follows: for control minimum = 89 mEPSCs, maximum = 1,010 mEPSCs; NE: 166–1013 mEPSCs; 5-HT: 77–809 mEPSCs. n.s., not significant; *significant difference.

To elucidate the physiological basis of monoamine modulation in the AVCN, we performed whole-cell voltage-clamp recordings to capture mEPSCs from BCs in brainstem slices of C57BL/6 wild-type mice in 2 mM Ca^2+^ supplemented aCSF during the third postnatal week as described in the Section 2. The mEPSCs recorded in the presence of exogenously added NE (100 μM) or 5-HT (10 μM) were compared to control data (Figure 4). We obtained recordings from 20 cells exposed to NE for 5 min (Figures 4A, B). Our control dataset consisted of mEPSC from 21 cells in the absence of exogenously added monoamines. We observed a significantly increased frequency of spontaneous release (Figure 4B) in response to NE exposure (7.85 ± 0.99 Hz, 8,538 mEPSCs, *n* = 20 cells from 9 mice, *p* = 0.0489) compared to control recordings (5.37 ± 0.88 Hz, 5,414 mEPSCs, *n* = 21 cells from 14 mice). In a similar manner, we recorded from 16 cells (7 mice) that were incubated with a 5-HT containing solution for 5 min (Figures 4A, B). Bath application of 5-HT did not result in significantly different mEPSC frequency compared to the control (5.02 ± 0.77 Hz, 4,821 mEPSCs, *p* = 0.9511). The amplitude and kinetic parameters of the eEPSCs remained unaltered in both cases of exogenously applied neuromodulators (data not shown).

**Figure 5:**
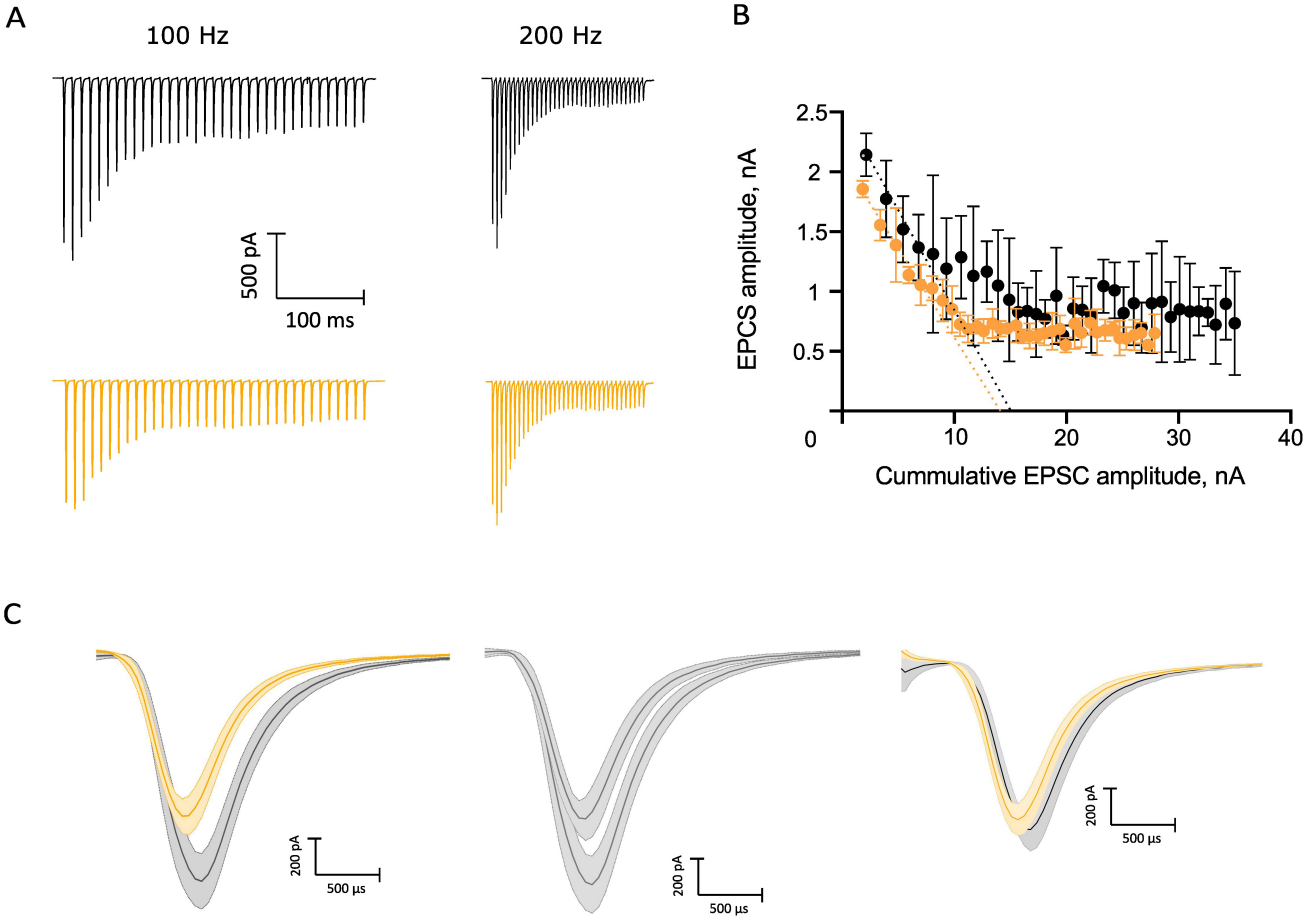
Probing for potential neuromodulation of evoked synaptic transmission. **(A)** Average traces of 100 and 200 Hz trains, control recordings in black, and NE recordings in orange. **(B)** Elmqvist–Quastel plot of the 100 Hz NE and control trains of an example cell. **(C)** Rundown over time, mean values of the first EPSC amplitudes (*n* = 3): left, comparison of the NE and control EPSC recordings of the 100 Hz trains used for the EQ plot in **(B)**; center, comparison of the control recordings in the beginning and end of the sampling of the control condition with a 59.3% difference; right, comparison between the recording following NE application and the control recording right before NE application. Scale bar 200 pA, 500 μs.

To study the effect of NE on evoked synaptic transmission at the endbulb of Held synapse, we used a monopolar electrode to elicit trains of 35 monosynaptic EPSCs (eEPSCs) in BCs of p15–19 mice by extracellular stimulation of individual endbulbs. We used two stimulation frequencies, 100 Hz, for which we obtained recordings from 18 BCs from 13 mice of which a subset of 16 BCs of 11 mice also provided EPSC train data in response to stimuli delivered at 200 Hz (Figure 5A). We observed a continuous decline in the amplitude of the eEPSCs at the different timepoints of recording under control conditions which we attribute to rundown of synaptic transmission. This can be appreciated from the difference in the amplitudes of the first eEPSCs (eEPSC_1_) of 100 Hz trains at the beginning and the end of the control condition (Figure 5C, middle) showing a reduction to 59.3% of the initial eEPSC_1_ amplitude. Furthermore, we compared the standard deviations (SD) of the eEPSC1 amplitude for each 3 repeats in the beginning and end of the control recording and they were significantly different (SD_beginning_: 0.26 nA, SD_end_: 0.13 nA, *p* = 0.0026). However, the SD between the amplitudes at the end of the control recordings and NE recordings was comparable (SD_NE_: 0.15 nA, *p* = 0.66). Because of this rundown, we compared the last three recordings before and first three recordings after applying 100 μM NE (Table 3, Figure 5C right). The amplitude and kinetics of the eEPSC_1_ appeared unaltered upon NE application. The synaptic delay did not reach statistical significance. Next, we investigated the properties of the 100 Hz and 200 Hz trains. For both frequencies, the kinetics of the stereotypic endbulb short-term depression reported by the time constant [Tau (τ), Table 4] of EPSC amplitude decay during train stimulation appeared unaltered by NE. The PPR both at 100 Hz and 200 Hz in the control and NE groups were comparable and often slightly facilitating in the beginning of the train. Because of the slight facilitation, we used the Elmqvist and Quastel (EQ) method (Figure 5B; Elmqvist and Quastel, 1965) to estimate the readily releasable pool (RRP) and P_vr_. We did not observe differences in P_vr_ between the control and NE datasets (Table 4). The RRP of the NE dataset tended to be smaller than in control (likely reflecting the described rundown) without reaching significance (Table 4). Using a subset of our data, we compared the above-mentioned parameters for 100 and 200 Hz recordings right before and after NE application (*n* = 6), to account for the rundown and observed no significant shifts (Supplementary Table S1). In addition, we wanted to explore neuromodulation in the context of higher variability of endbulb transmission. For this purpose, we lowered the Ca^2+^ concentration to 1.3 mM. However, we did not uncover any changes in the parameters of EPSCs at 100 Hz (*n* = 4 cells) and 200 Hz (*n* = 3 cells) (Supplementary Table S2). In summary, we observed an increase on the frequency of spontaneous release upon NE application, but no effect on evoked release was detected.

**Table 3:** Comparison of the first EPSC parameters before and after 100 μM NE application.

**1^st^ EPSC parameter (18 cells)**	**Control**	**NE**	***p*-value**
Amplitude, nA	1.19 ± 0.11	1.14 ± 0.11	0.79
Synaptic delay, ms	1.09 ± 0.05	0.97 ± 0.05	0.09
Rise time, ms	0.20 ± 0.01	0.22 ± 0.03	0.57
Decay time, ms	0.40 ± 0.02	0.37 ± 0.02	0.39

**Table 4:** Comparison of parameters of trains of EPSCs at 100 and 200 Hz from the control and NE datasets.

**Frequency**	**Parameter**	**Control**	**NE**	***p*-value**
100 Hz	Pvr_(EQ)_	0.14 ± 0.01	0.13 ± 0.01	0.80
	RRP_(EQ)_, SVs	396.88 ± 50.89	276.38 ± 34.24	0.06
	PPR	1.07 ± 0.09	1.04 ± 0.05	0.86
	τ of depression, ms	44.98 ± 2.72	41.93 ± 2.68	0.44
200 Hz	Pvr_(EQ)_	0.20 ± 0.01	0.17 ± 0.02	0.12
	RRP_(EQ)_, SVs	259.43 ± 30.46	247.78 ± 25.01	0.78
	PPR	1.16 ± 0.05	1.20 ± 0.06	0.62
	τ of depression, ms	19.85 ± 0.88	20.93 ± 1	0.44

### 3.4 Testing for noradrenergic or serotonergic modulation of BC excitability

**Figure 6:**
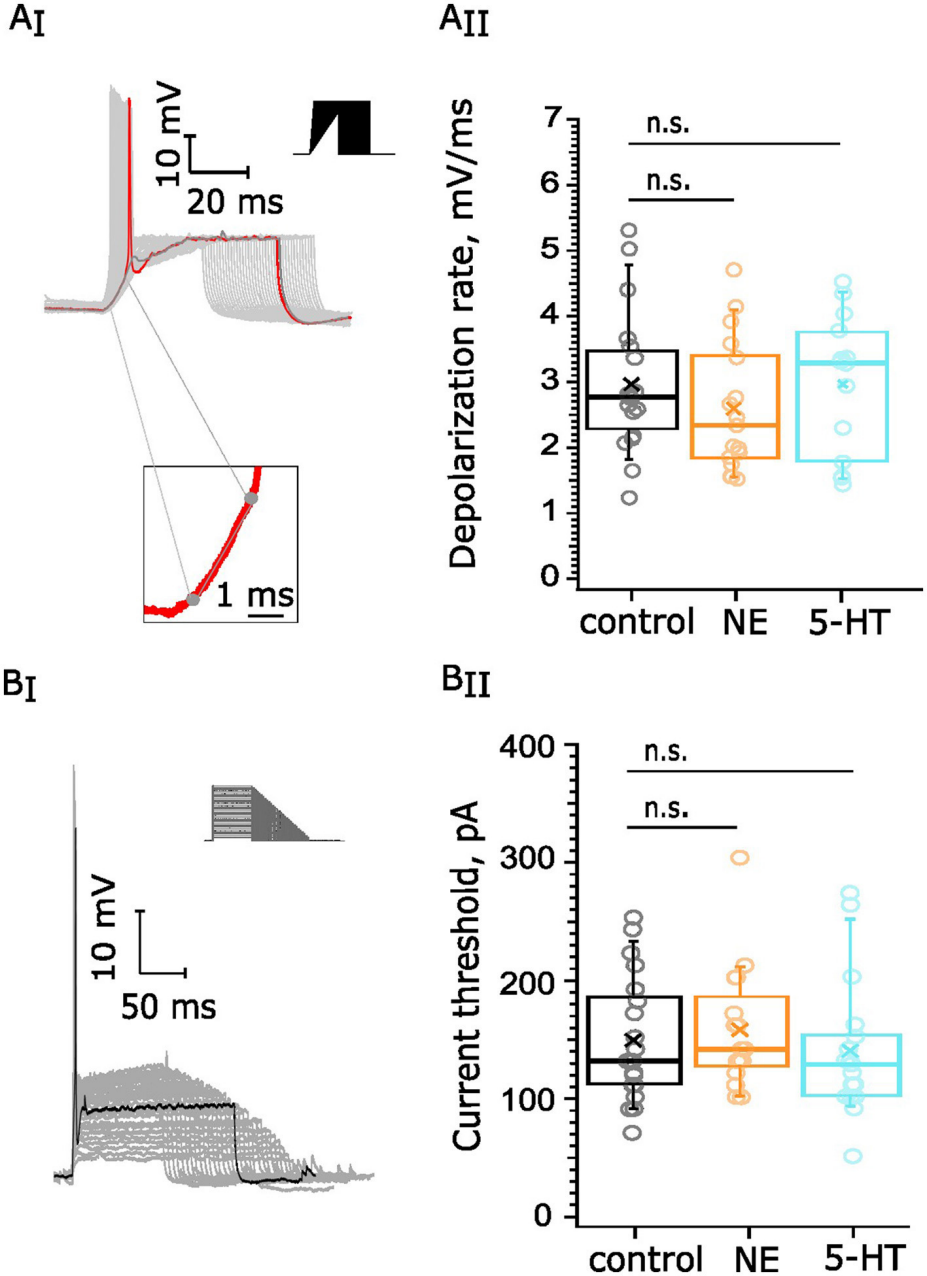
Testing for neuromodulation of BC excitability. 10 μM 5-HT or 100 μM NE were applied for 5 min following recordings under control conditions: **(A)** BCs' rate of depolarization, 19 cells (14 mice) in the control dataset, 14 cells (7 mice) for 5-HT, and 17 cells (7 mice) for NE. **(A**_**I**_**)** We documented the slowest rate at which an AP is generated (red trace) and estimated the derivative of the linear component of the voltage trace before the generation of the AP. Scale bar 10 mV, 20 ms. **(A**_**II**_**)** The rate of depolarization was not altered upon NE (*p* = 0.29) or 5-HT (*p* = 0.93) administration. **(B)** Current thresholds of BCs firing. Our datasets consisted of recordings from 20 cells, 13 mice in the control dataset, 17 cells, 7 mice for 5-HT one, and 16 cells, 7 mice for NE. Scale bar 10 mV, 50 ms. **(B**_**I**_**)** We injected currents with different amplitudes and duration (top right corner) and documented the first current value at which an AP (black) was generated. **(B**_**II**_**)** The current threshold did not display significant change upon NE application (*p* = 0.5772 or upon 5-HT administration, *p* = 0.4734). n.s., not significant.

We next probed for potential effects of NE and 5-HT on the excitability of the BC. BCs are sensitive to the speed of depolarization due to their g_KL_, and we investigated whether neuromodulation could tune this sensitivity. For this purpose, we subjected BC to currents with different rates of depolarization in current clamp experiments (Figure 6 A_I_). We documented the slowest rate at which an AP is generated and compared it between experimental groups. Our collected datasets consisted of recordings from 19 cells from 14 mice in the control condition, 14 cells from 7 mice in the 5-HT group (10 μM), and 17 cells from 7 mice exposed to NE (100 μM). The average rate of depolarization in the NE dataset was 2.60 ± 0.24 mV/ms (Figure 6 A_II_), which was statistically indistinguishable from the depolarization rate of control recordings (*p* = 0.29). The average rate of depolarization appeared to be quite similar between the control (2.97 ± 0.24 mV/ms) and 5-HT groups (2.99 ± 0.27 mV/ms, *p* = 0.93, Figure 6A_II_).

Next, we aimed to examine whether the excitability of the BCs was altered upon NE or 5-HT application. For this purpose, we quantified the current threshold for AP generation—the minimal current amplitude of infinite duration that leads to the crossing of the depolarization threshold of the cell membrane (Figure 6B). We obtained a dataset of control recordings from 20 cells among 13 mice as well as recordings from cells, exposed for 5 min to 5-HT (17 cells, 7 mice) or NE (16 cells, 7 mice). The average current threshold of the control recordings was 149.52 ± 11.54 pA. Similarly, the current threshold of the NE recordings (158.4 ± 12.7 pA, *p* = 0.5772, Figure 6B_II_) and the current threshold of the 5-HT recordings (139.9 ± 13.8 pA, *p* = 0.4734, Figure 6B_II_) showed similar values in comparison with the control. In conclusion, neither 100 μM NE nor 10 μM 5-HT affected depolarization speed and current threshold of BCs.

In addition, we delivered trains of action potentials by presynaptic stimulation of the BCs at 100 Hz and 200 Hz in current clamp. We did not observe significant shifts in the first AP kinetics, the spike probability at 200 Hz or the amplitude of the APs over the course of the 100 Hz trains for both 5-HT and NE (Supplementary Figures S3–S6).

### 3.5 Probing for noradrenergic or serotonergic modulation of outward currents of BCs

**Figure 7:**
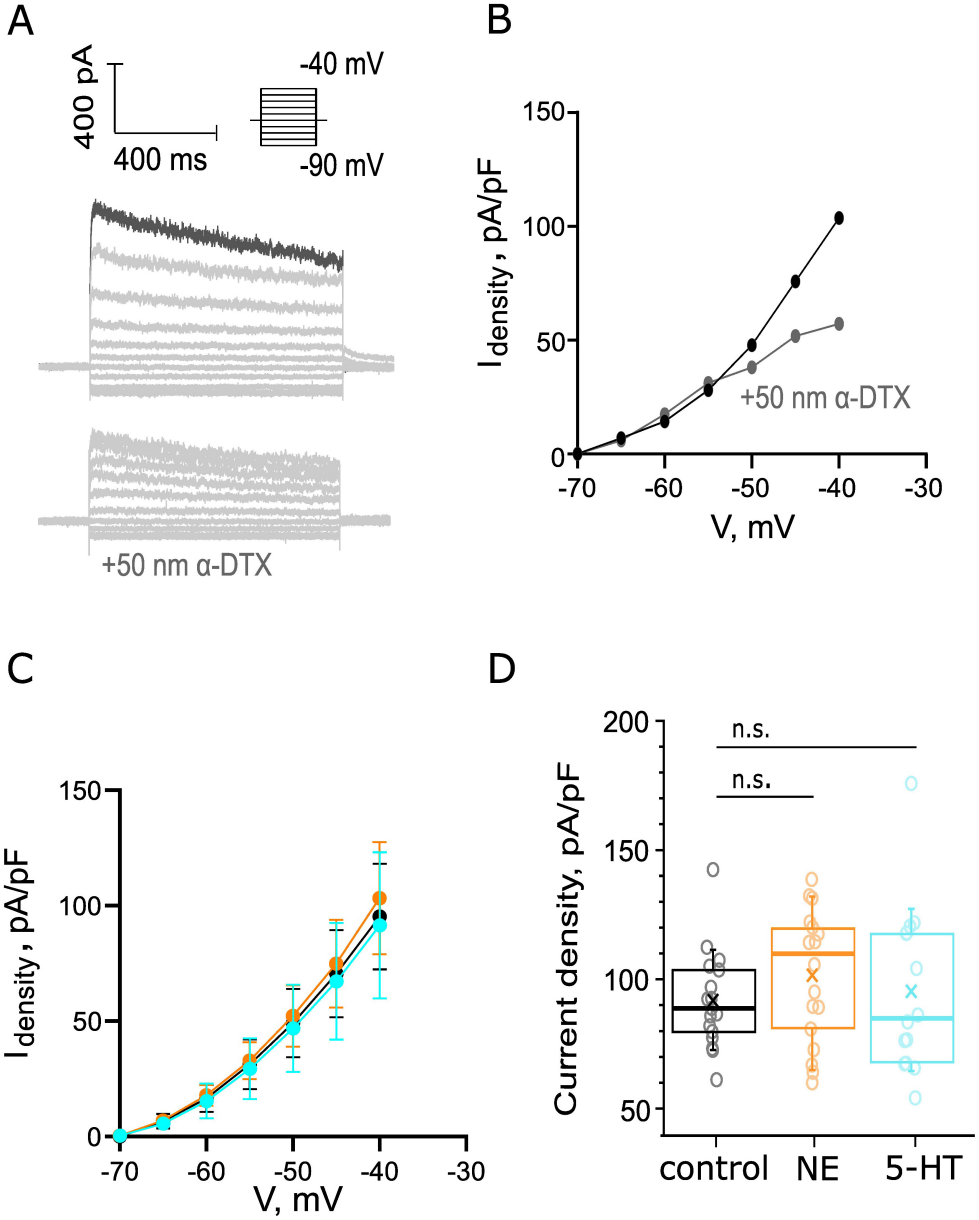
Probing for noradrenergic or serotonergic modulation of outward currents of BCs. **(A)** An example recording of positive outward current in BC. Scale bar 400 pA, 400 ms. Depolarizing voltage steps (5mV) from −90 mV to −40 mV activated voltage-sensitive outward current. Top: Recordings were made in the presence of 10 μM ZD7288, 1 μM TTX, 0.25 mM CdCl_2_, 10 μM DNQX, 2 μM strychnine, and 10 μM bicuculline. Bottom: a negative control for I_KL_, addition of 50 nM α-DTX to the solution reduced the outward current at −40 mV with 44.87%. The current density was calculated as the ratio between the measured current at −40 mV of each recording and the C-slow capacitance of each cell. **(B)** IV curve before (black) and after (gray) the perfusion of 50 nM α-DTX (gray). **(C)** The IV curves of the control (*n*_control_ = 17 cells from 8 mice, black), NE (*n*_NE_ = 18 cells from 7 mice, orange), and 5-HT (*n*_5 − HT_ = 14 cells, 7 mice, cyan) datasets from −40 to −70 mV. **(D)** The peak current density was not significantly affected by NE perfusion (*p* = 0.2017). The peak current density was unaltered by 5-HT perfusion (*p* = 0.9443).

Next, we probed for potential effects of NE and 5-HT on the currents mediated by low-voltage-activated K^+^ channels (I_KL_, reference, Materials and Methods). We applied depolarizing voltage steps (5 mV) from −90 to −40 mV activating voltage-sensitive outward current and added blockers of to isolate K^+^ currents (10 μM ZD7288, 1 μM TTX, 0.25 mM CdCl_2_, 10 μM DNQX, 2 μM strychnine, and 10 μM bicuculline). The peak of the I_KL_ positive currents has been shown to be at −40 mV (Fu et al., 2021), so we focused on currents recorded at −40 mV (Figure 7A top, darker trace) for our analysis. To estimate what portion of this peak outward current densities at −40 mV is attributed to I_KL_, we applied α-dendrotoxin (α-DTX, 50 nM) which blocks Kv_1.1_, Kv_1.2_, and Kv_1.6_ channels. This solution was perfused for 9 min following control recordings. We observed a 44.87% reduction of the outward current densities at −40 mV (Figures 7A bottom, B). We then probed for potential effects of application of 100 μM NE and 10 μM 5-HT, respectively. We did not detect significant differences in the IV curves of our group datasets upon application of either neuromodulator (Figure 7C). The average outward current density (101.6 ± 5.7 pA/pF from 18 cells, 7 mice, *p* = 0.2017, Figure 7D) at 5 min of 100 μM NE perfusion tended to be increased compared to the density of the control dataset (91.0 ± 4.4 pA/pF from 17 cells, 8 mice) without reaching statistical significance. The average current density of the control and 5-HT datasets was comparable: 5-HT dataset: 91.1 ± 8.2 pA/pF from 14 cells, 7 mice, *p* = 0.94 (Figure 7D). In conclusion, we did not observe changes in the I_KL_ peak current density while pharmacologically applying 10 μM 5-HT or 100 μM NE.

### 3.6 Probing for noradrenergic or serotonergic modulation of hyperpolarization-activated currents of BCs

**Figure 8:**
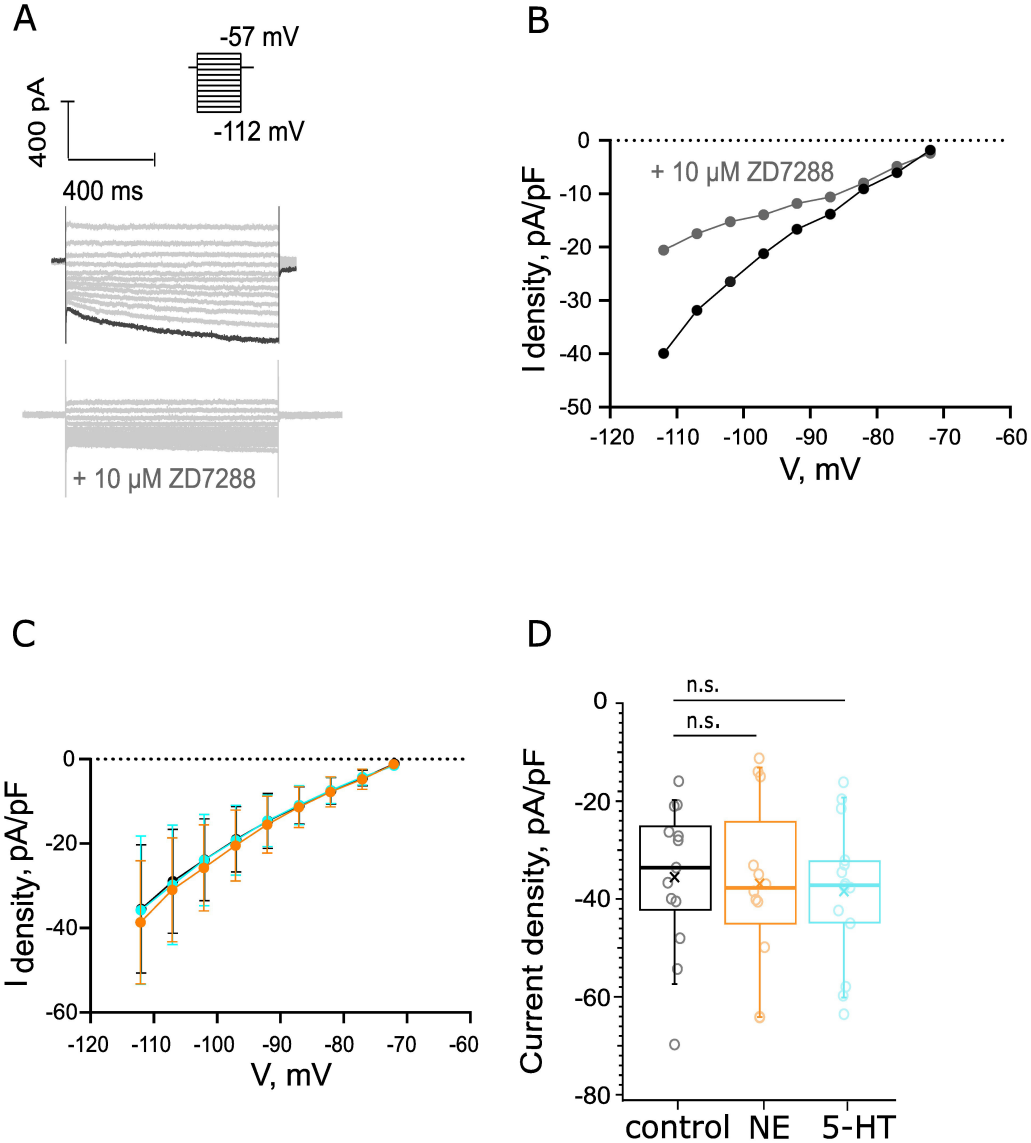
Probing for noradrenergic or serotonergic modulation of hyperpolarization activated currents of BCs. **(A)** Voltage steps (5 mV) from −112 to −57 mV activated voltage-sensitive inward current at the hyperpolarizing voltages. Scale bar 400 pA, 400 ms. Top: the recordings were made in the presence of 25 nM α-DTX, 1 μM TTX, 0.25 mM CdCl_2_, 10 μM DNQX, 2 μM strychnine, and 10 μM bicuculline. Bottom: negative control for I_h_ currents, addition of 10 μM ZD7288 reduced the inward currents with 48.49%. The amplitude of the inward current that we measured was derived as the average current amplitude at the last 100 ms of the voltage trace recorded at −112 mV. The current density was calculated as the ratio between the measured current at −112 mV of each recording and the C-slow capacitance of each cell. **(B)** IV curve before (black) and after (gray) the perfusion of 10 μM ZD7288. **(C)** The IV curves of the control (n_control_ = 13 cells from 7 mice, black), NE (*n*_NE_ = 12 cells from 5 mice, orange), and 5-HT (*n*_5 − HT_ = 14 cells, 6 mice, cyan) datasets from −72 to −112 mV. **(D)** The current density was unaltered in the presence of 100 μM NE after 5 min of perfusion (*p* = 0.96). We did not observe a significant difference in the current density in the presence of 10 μM 5-HT at 5 min of perfusion (*p* = 0.58).

Finally, given the immunohistochemical indication of the presence of α_2C_-AR, 5-HT_1B_R, 5-HT_4_R, and 5-HT_7_R in BCs which modulate cAMP levels, we decided to probe for effects of NE or 5-HT on hyperpolarization induced (I_h_) currents that are modulated cAMP. Voltage steps (5 mV) from −112 mV to −57 mV activated voltage-sensitive inward current at the hyperpolarizing voltages in the presence of 25 nM α-DTX, 1 μM TTX, 0.25 mM CdCl_2_, 10 μM DNQX, 2 μM strychnine, and 10 μM bicuculline (Figure 8A). Our recorded traces were sorted in the following groups: a control group, an NE group, comprising recordings from cells exposed to 100 μM NE for 5 min, and finally a 5-HT group, subjected to 5 min of bath-applied 10 μM of 5-HT. To estimate what portion of this negative current is attributed to I_h_, we performed a negative control. First, we recorded the outward current using our control solution, which was followed by perfusion of a solution, supplemented with 10 μM ZD7288 to block HCN channels. This second solution was perfused for 9 min, and the difference in currents can be appreciated in Figures 8A, B. We observed a 48.49% reduction of the inward current at −112 mV, which could be attributed to the blocking of HCN channels. The IV curves of the negative currents in our three datasets are depicted in Figure 8C. The average inward current density estimated as the plateau at −112 mV of control dataset recordings (−35.48 ± 4.05 pA/pF from 13 cells, 7 mice) was comparable to that yielded from both the NE (−35.79 ± 4.85 pA/pF from 12 cells, 5 mice, *p* = 0.6280, Figure 8D) and 5-HT (−38.7 ± 3.77 pA/pF from 14 cells, 6 mice, *p* = 0.58, Figure 8D) datasets. To conclude, we did not detect changes in I_h_ upon administration of 10 μM 5-HT or 100 μM NE.

## 4 Discussion

In this study, we provide initial morphological and functional evidence for monoaminergic innervation of the CN. We found varicose neurites featuring dense core and synaptic vesicles in volume EM reconstructions made for the AVCN and DCN which are compatible with monoaminergic innervation. Moreover, we obtain immunohistochemical proof of monoamine transporters being present in the CN and explored the receptor complement of such neurotransmitters. Our report of expression of a_2C_-AR as well as 5-HT_7_, 5-HT_1B_, and 5-HT_4_ receptors in the AVCN provides support for volume neuromodulatory transmission affecting this early auditory circuit. Finally, we found a subtle increase in the mEPSC frequency of BCs upon application of 100 μM NE suggesting a direct noradrenergic modulation of the endbulb of Held. Yet, much remains to be done to rigorously analyze the functional role of monoaminergic innervation of the CN beyond this first preliminary study.

### 4.1 Morphological evidence for the presence of varicosity-containing projections in the cochlear nucleus

In this study, we provided immunohistochemical evidence for the presence of the NET and SERT in the vicinity of the endbulb-BC synapse. To further consolidate this finding of monoamine releasing varicosities, we turned to SBEM and found varicosity-like neurites in the DCN and the AVCN. Indeed, a previous electrophysiological study in the mouse DCN demonstrated serotonergic innervation acting through 5-HT_2_ and 5-HT_7_ receptors (Tang and Trussell, 2015). Further studies should extend the volume EM analysis to larger volumes and also employ immuno-EM experiments to test the correspondence of these varicosities to 5-HT neurons. Our preliminary investigation of monoamine receptors in the AVCN revealed initial evidence of 5-HT_1B_, 5-HT_4_, and 5-HT_7_ receptors and the α_2C_-adrenergic receptors inside BCs and near their plasma membrane. The intracellular localization of the receptors could be explained by GPCR internalization as a result of stimulation. This mechanism of action has been described for the 5-HT_2A_R, where after stimulation, the GPCR colocalizes with endosome markers such as Rab5 and Rab7 (Eickelbeck et al., 2019). In addition, we observed 5-HT_7_R and α_2C_-AR signal in the vicinity of the endbulbs in some of the BCs that we imaged. However, the use of higher resolution imaging, such as STED microscopy, will be required to further and more accurately confine the localization of the receptors relative to pre- or postsynaptic elements.

The 5-HT_7_R is coupled to G_s_ proteins and its activation raises cAMP levels which in turn trigger PKA-dependent effector cascades (Bard et al., 1993; Lovenberg et al., 1993; Ruat et al., 1993). The 5-HT_7_ receptor is robustly expressed in the thalamus and hypothalamus, as well as the hippocampus and cortex. This receptor is involved in thermoregulation, circadian rhythm, learning and memory, sleep, and mood regulation (Hedlund and Sutcliffe, 2004). Similarly, the 5-HT_4_R is a G_s−_coupled receptor (Dumuis et al., 1988; Bockaert et al., 1992) that is involved in learning, memory, and mood regulation and is expressed in the cortex and hippocampus, as well as limbic regions, namely in the basal ganglia and amygdala (Bockaert et al., 2006). On the contrary, the 5-HT_1B_R is coupled to G_i_ proteins (Bouhelal et al., 1988) and expressed in the cortex and basal ganglia (Bockaert et al., 2006). We observed the 5-HT_1B_R signal in our staining in structures localizing near BC. This suggests that other cell types are regulated by 5-HT signaling in the AVCN. Yet, complimentary electrophysiological experiments will be required to elucidate the mechanism of action of 5-HT_7_, 5-HT_4_, and 5-HT_1B_ receptors in BC.

α_2C_-ARs are known to serve both as autoreceptors, controlling noradrenaline release, and as heteroreceptors. α_2C_-AR heteroreceptors were shown to regulate 5-HT transmission by inhibition of 5-HT release, but to a lesser extent than α_2A_-AR (Scheibner et al., 2001). Concentrations of NE and 5-HT were shown to be increased in the brains of α_2C_-AR knock-out mice (Sallinen et al., 1997). In this context, the presence of the α_2C_-AR in the vicinity of the endbulb synapse may indicate an intricate regulation network of this early integration station of the auditory pathway. As for 5-HT_7_ receptors, further work is required to dissect the mechanism of action of 5-HT in the AVCN.

In addition, other receptor subtypes might be present, but we might have failed to detect them under the chosen conditions and with the commercial antibodies at our discretion. A way to tackle this could be the use of melanopsin variants called CaMello-XR (Eickelbeck et al., 2019). These are G-protein-coupled opsins, carrying a fluorescent label (e.g., mCherry or eGFP). The C-terminus of these proteins can be modified to contain an amino acid sequence from the C-terminal region of a neuromodulator receptor (e.g., 5-HT_2A_), which is a localization signal that leads to the trafficking of the protein to the membrane domains that are normally occupied by the known receptor.

### 4.2 Neuromodulator pharmacology and synaptic transmission in the AVCN

We observed an augmented mEPSC frequency in the presence of 100 μM NE which reflects a higher number of SV spontaneously fusing with the membrane. Potentially, this could be attributed to the priming of synaptic vesicles and transitions from lose to tight docking state (Neher and Brose, 2018), further explainable by an overall increase of available vesicles at AZs (Patzke et al., 2019), yet without affecting the kinetics of state transitions. However, to argue in favor of such speculations, we need mechanistic proof for such a process. Our evoked release data did not show significant shifts in the PPR, P_vr_, and RRP, nor in the amplitude of the EPSCs or the τ of depression. There are indications for differential regulation of spontaneous and evoked release (Ramirez and Kavalali, 2011). Hence, there is a possibility that the increased frequency of spontaneous release upon NE application is governed by a regulation mechanism separate from the ones controlling evoked release. In addition, we did not uncover a significant shift in the BCs' characteristic ion conducting channels. It is possible that these channels are in fact not regulated by neuromodulators and/or that neuromodulation operates on AVCN processes that we did not analyze. A recent study revealed that cAMP reduced the amplitude of APs and the speed of their propagation over long axonal distances due to sodium current inhibition (Abate et al., 2024). This could potentially serve as a tuning mechanism of transmission between the SGNs and BCs. However, in our preliminary AP train experiments with a dataset of five cells, we did not observe a significant change is APs' size over the course of the train. Still, the non-physiological pharmacological application of monoamines, the rundown of synaptic transmission during the long recordings, and/or the limited size of the datasets might have prevented detection of neuromodulatory effects.

## 5 Limitations of the present study

A clear limitation of the wash-in experiments is the possibility of receptor desensitization. To further probe for monoaminergic modulation of the endbulb synapse, more specific stimulation or inhibition of endogenous monoaminergic modulation of the AVCN should be used. This could be achieved through optogenetic control of the putative monoamine projections in the AVCN. In future studies, we aim to expand our work in this direction through AAV-mediated cell-type specific expression of excitatory and inhibitory ChRs using appropriate Cre mice such as the Dbh-cre and SERT-cre lines. Furthermore, the subtle effect of NE of spontaneous release should be verified by wash-in experiments in single cells, enabling before–after comparisons. The high concentration of EGTA used in our intracellular solution might buffer Ca^2+^-induced responses and influence the active membrane properties, particularly the resting membrane potential, depending on the contribution of Ca^2+^ sensitive K^+^ channels. In addition, the AVCN is a complex structure, and thus, we cannot rule out the possibility that synapses other than the one we studied could be undergoing neuromodulation. Potential effects of the whole-cell patch-clamp configuration of exogenously added intracellular Ca^2+^ buffering on the postsynaptic currents or excitability of bushy cells could be avoided by future perforated-patch recordings. Furthermore, our immunohistochemistry data need to be backed up with functional evidence for the action of NE and 5-HT at the endbulb-BC synapse. In our EM study, we uncovered neurites that could potentially be of monoaminergic origin; however, this is yet to be confirmed. Perhaps post-embedding immunogold EM or even Freeze-fracture Replica Immunogold Labeling (FRIL) using robust antibodies against surface molecules such as SERT or NET could elucidate the precise localization of noradrenergic neurites relative to known AVCN circuit elements.

## Data Availability

The original contributions presented in the study are included in the article/Supplementary material, further inquiries can be directed to the corresponding authors. The SBEM raw data supporting Figure 1 is now publicly accessible via https://wklink.org/7813.
