# Supplementary material for: Investigation of neuromodulation of the endbulb of Held synapse in the cochlear nucleus by serotonin and norepinephrine
Source: Front Cell Neurosci. 2025 Apr 28;19:1575158. doi: 10.3389/fncel.2025.1575158 (PMC12066487; doi:10.3389/fncel.2025.1575158)
Supplement: Supplementary file 1 [file Data_Sheet_1.docx]

# Supplement

S1 Supplementary methods – optimization of the staining protocol

S2 Positive control immunostainings of the locus coeruleus and Raphe nuclei

S3 Evaluating the action of 5-HT on the parameters of the first AP at 100Hz stimulation frequency

S4 Evaluating the action of NE on the parameters of the first AP at 100Hz stimulation frequency.

S5 Normalized amplitude of the BC last AP resulting from 100 Hz train stimulation

S6 BC spike probability (SP) at 200 Hz

S7 Comparison of parameters of trains of EPSCs at 100Hz and 200 Hz from the control and NE datasets measured at 1.3 mM Ca2+.

S8 Comparison of parameters of trains of EPSCs at 100Hz and 200 Hz from the control and NE datasets measured right before and after NE application.

## Supplementary methods – optimization of the staining protocol

Our survey started with a parallel probing for β_3_-AR (S1, G) and 5-HT_2A_R (S1, C) using the primary antibodies against these receptors at a dilution of 1:200. The receptors in these cases were marked with a secondary Ab Alexa Fluor 568, while the markers V-Glut1(1:1000) – with Alexa Fluor 488 and Gephyrin (1:500) – with Alexa Fluor 633. We acquired z-stacks of each BC and presented them as maximum intensity projections.

What can be readily observed in these experiments is strong bleed-through from the 488 nm channel to the 568 nm one. Apart from the bleed-through overlap, according to the negative controls the signal representing the receptors of interest showed a small grainy dotted pattern localized both inside and around the cells. Additionally, the signal from the 633 nm channel was not entirely specific for Gephyrin, but also delineated blood vessels (unspecific binding).

Consequently, we performed stainings against β_1_-AR (S1, E), β_2_-AR (S1, F), 5-HT_1A_R (S1, B) and 5-HT_5B_R (S1, A) in dilution 1:200 and using a secondary Ab with peak emission 488 nm wavelength, the marker vGlut1 – with peak at the 568 nm wavelength and using a different secondary Ab against gephyrin, this time conjugated with Alexa Fluor 633 nm. However, our Gephyrin staining was still unsuccessful. Our staining against the 5-HT_5B_R displayed a strong signal and no bleed-though. In the stainings against the β1-AR and 5-HT_1A_R, bleed-through was still present, even from the 568 nm to the 488 nm channel. The β2-AR staining showed localization of the Ab on the nuclear membrane.

We used the staining against β3-AR as a reference for further optimization. To avoid bleed-through we conjugated the anti-β3-AR Ab with Alexa Fluor 488 and vGlut1 – with Alexa Fluor 633. However, even in this configuration where the two markers were placed far from each other spectrally bleed-through was still present. We also tried to increase the anti-β3-AR Ab concentration to 1:100, due to the concern that bleed-through was more relevant for a faint signal. However, this did not improve the quality of the images but rather more unspecific binding occurred.

Therefore, we decided to decrease the concentration to 1:500 and probe for new receptors, to expand our dataset, since the studied ones so far might not display prominent or any expression in the vicinity of the EoH – BC synapse. Additionally, we decided to replace the postsynaptic marker Gephyrin with Homer1, because of the observed unspecific binding to capillaries. The new batch of receptors tested included 5-HR_7_R (Fig. 2 B, C), 5-HT_2B_R (S1, D), α_1B_-AR (S1, H), α_1D_-AR (S1, I) and α_2C_-AR (Fig. 2 A). The stainings against the 5-HR_7_R and α_2C_-AR receptors showed a high intrinsic signal and no bleed-through was observed in those probes. This led us to the conclusion that the specific immunofluorescence signal in the previous stainings displaying bleed-through (examples on Fig. S1) was too dim such that the high gain setting being used during image acquisition emphasized the background signal.


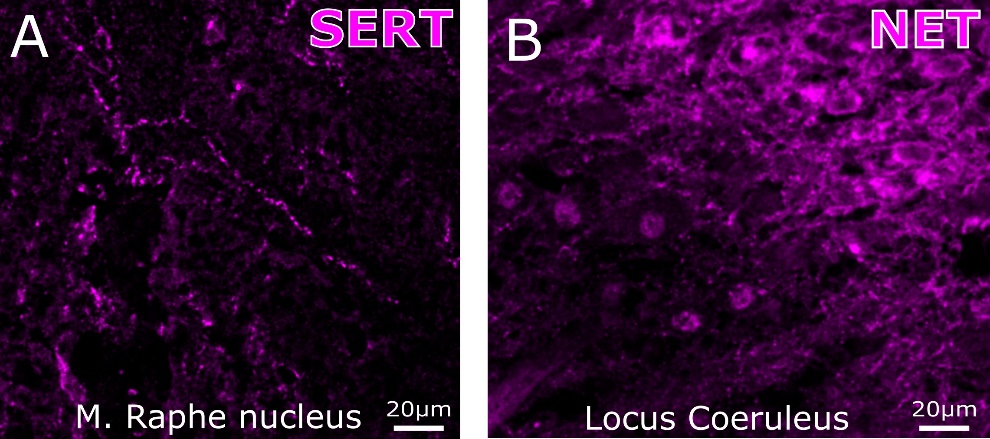


Figure S1 Positive control immunostainings of the locus coeruleus and raphe nuclei: A, NET staining in the LC. B, SERT staining in the M. RN.


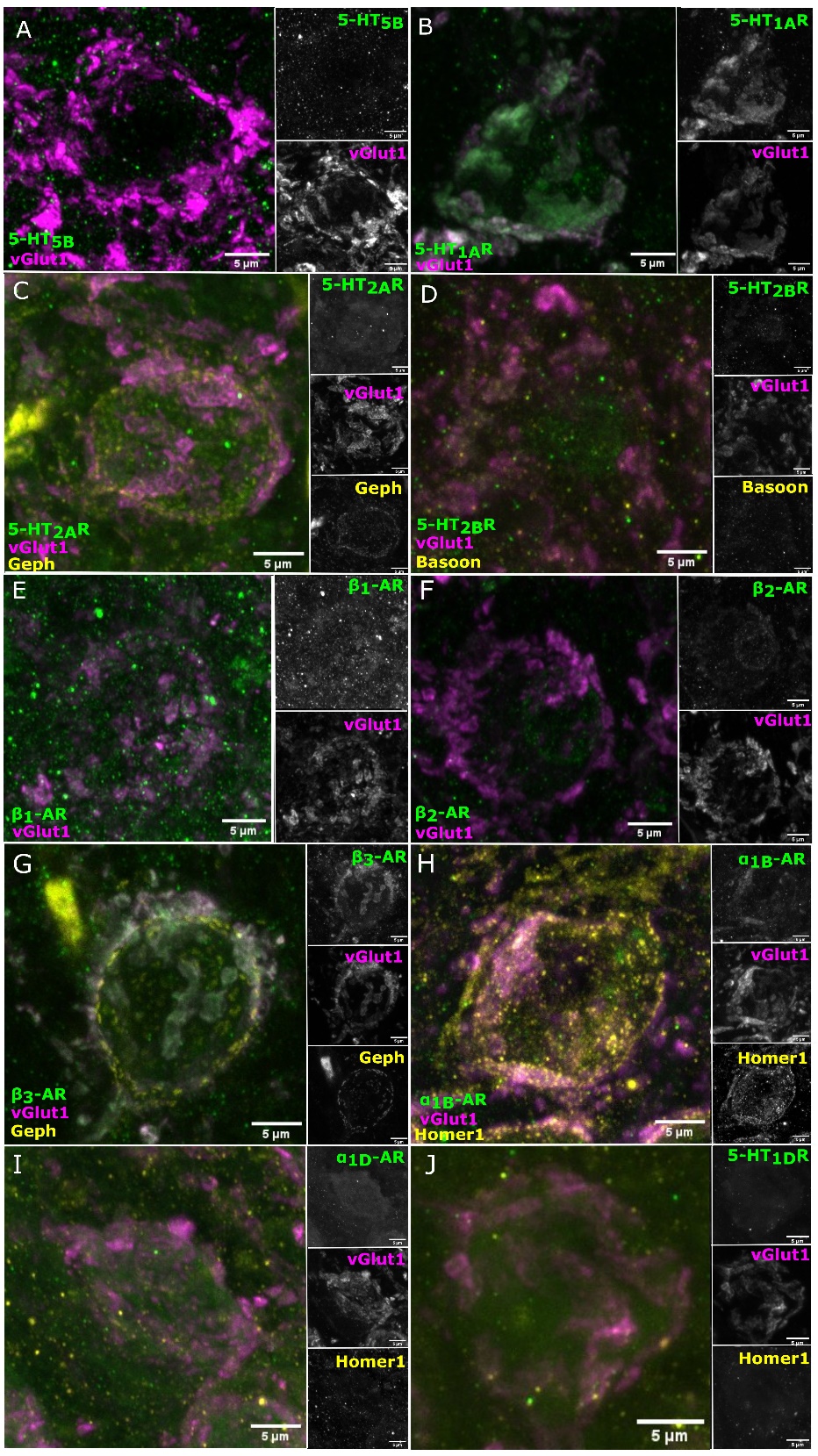


Figure S2 Stainings against 5-HT and NE receptors, optimization of the staining protocol. All antibodies are tested with a complimentary negative staining containing the binding peptide of the monoamine receptor AB (data not shown). A, 5-HT_5B_ receptor in green (Alexa-fluor-488), vGlut1 in magenta (Alexa-fluor-568). B, HT_1A_R in green (Alexa-fluor-488), vGlut1 in magenta (Alexa-fluor-568). C, 5-HT_2A_R in green (Alexa-fluor-488), vGlut1 in magenta (Alexa-fluor-568) and gephyrin in yellow (Alexa-fluor-633). D, 5-HT_2B_R (Alexa-fluor-488), vGlut1 in magenta (Alexa-fluor-568), bassoon in yellow (Alexa-fluor-633). E, β_1_-AR in green (Alexa-fluor-488), vGlut1 in magenta (Alexa-fluor-568). F, β_2_-AR in green (Alexa-fluor-488), vGlut1 in magenta (Alexa-fluor-568). G, β_3_-AR in green (Alexa-fluor-568), vGlut1 in magenta (Alexa-fluor-488) and gephyrin in yellow (Alexa-fluor-633). H, α_1B_-AR in green (Alexa-fluor-488), vGlut1 in magenta (Alexa-fluor-568) and Homer1 in yellow (Alexa-fluor-633). I, α_1D_-AR in green (Alexa-fluor-488), vGlut1 in magenta (Alexa-fluor-568) and Homer1 in yellow (Alexa-fluor-633). J, 5-HT_1D_R in green (Alexa-fluor-488), vGlut1 in magenta (Alexa-fluor-647) and Homer1 in yellow ((Alexa-fluor-568)


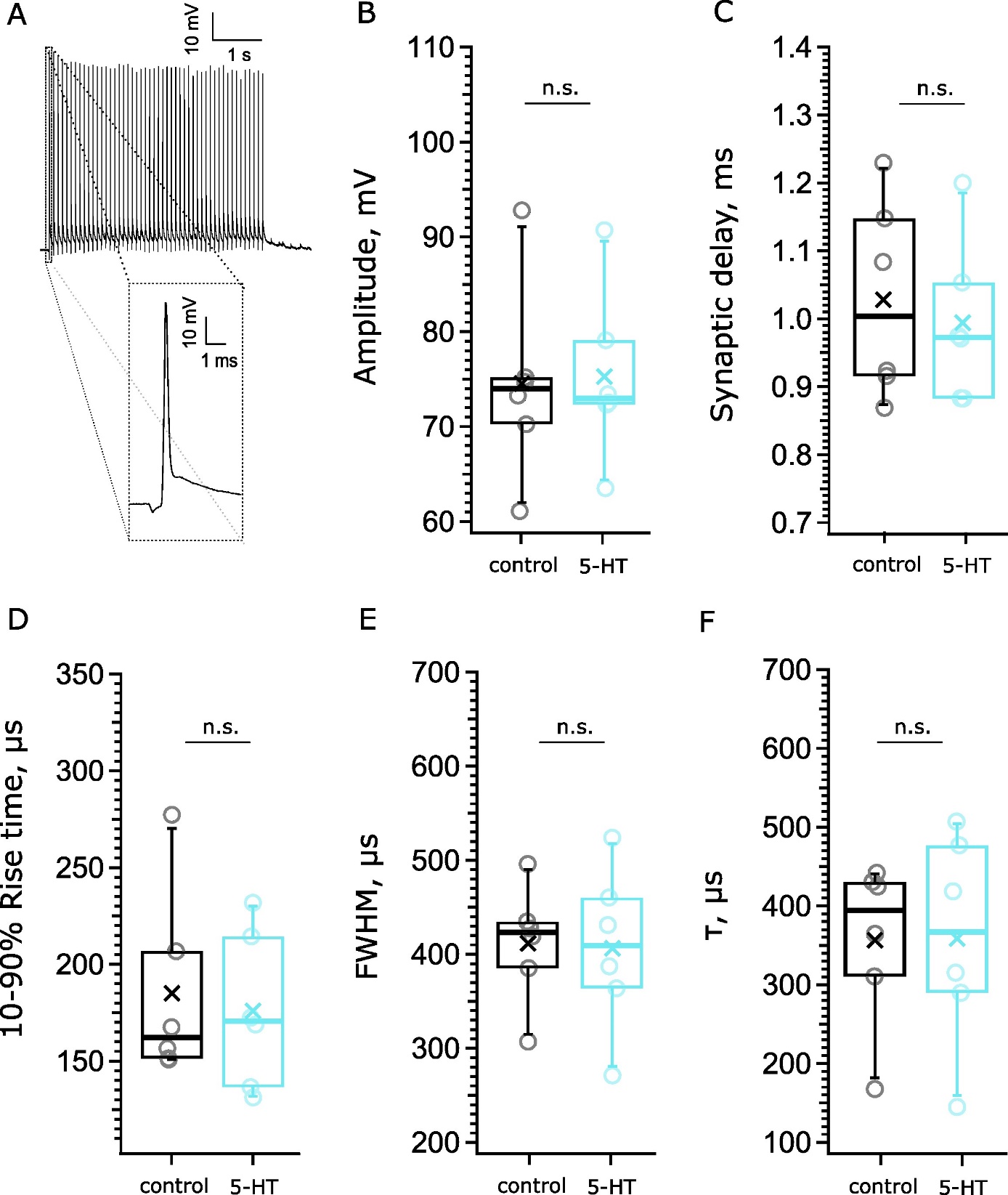


Figure S3 First action potential (AP) parameters at 100Hz stimulation frequency during 10 µM 5-HT application. We performed current-clamp recordings of BC action potentials (APs) in response to train stimulations delivered to the afferent ANFs with a monopolar electrode. We first acquired AP trains in the control solution, followed by recording responses to the same sequence of stimuli, while bathing the cells with the 100 µ NE or the 5-HT solution. N=6 cells from 6 mice A, An example recording, B, Amplitude of the AP, *p=0.5722*, C, Synaptic delay, *p=0.8815*. D, 10-90% rise time of the AP, *p=0. 7105*, E, Full width at half maximum (FWHM) of the AP, *p=0.9045,* F, decay of the AP (τ), *p=0.4670*, Each data point represents an average of the given parameter across the events in one mEPSC recording – black for the control recordings and orange for NE recordings.


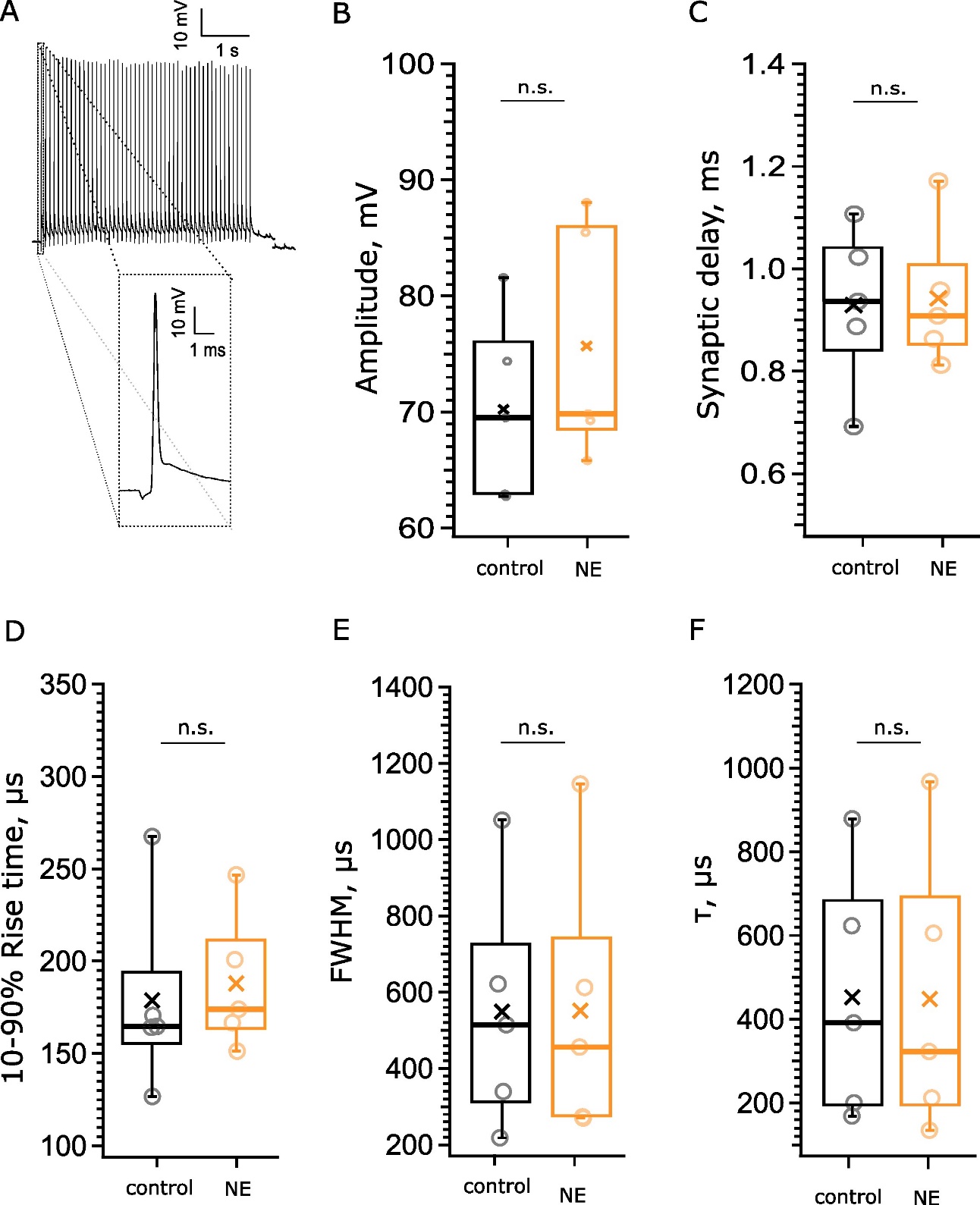


Figure S4 First action potential (AP) parameters at 100Hz stimulation frequency during 100 µM NE application. The acquisition protocol is described in Fig. S3. N= 5 cells from 4 mice. A, An example recording. B, Amplitude of the AP, p=0.3705. C, Synaptic delay, p=0.8178. D, 10-90% rise time of the AP, p=0.7590, E, Full width at half maximum (FWHM) of the AP, p=0.9908, F, decay of the AP (τ), p=0.9849, Each data point represents an average of the given parameter across the events in one mEPSC recording – black for the control recordings and orange for NE recordings.


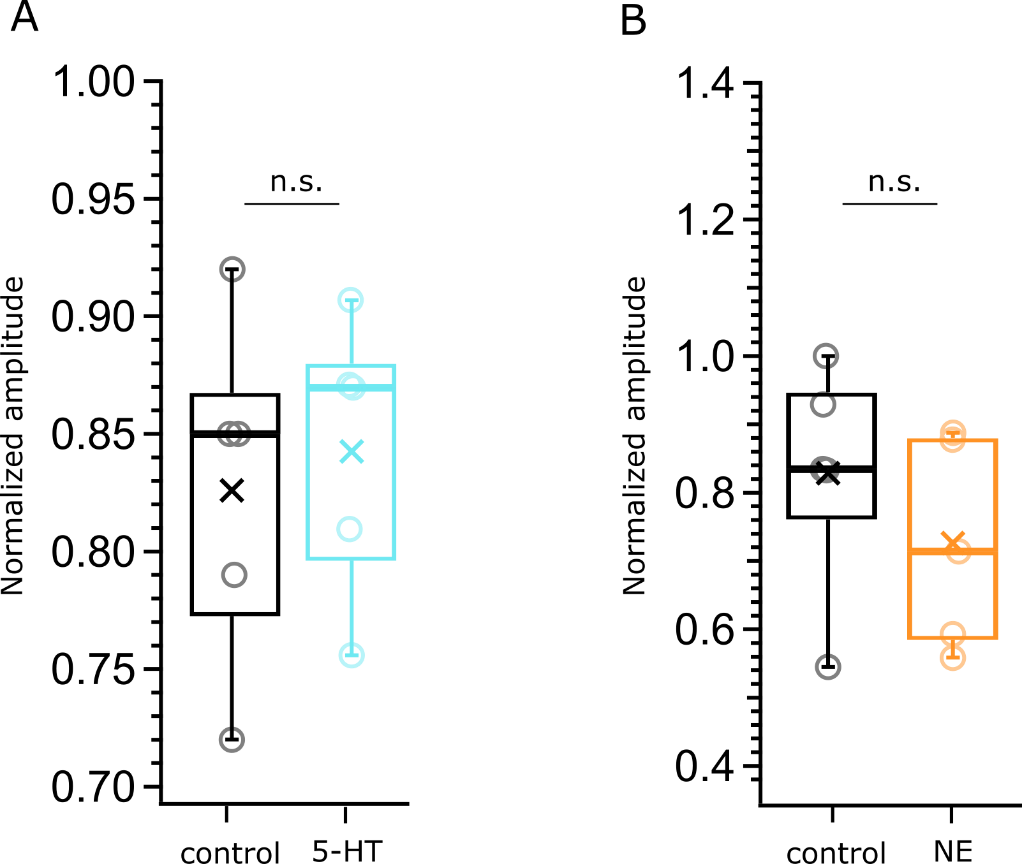


Figure S5 Normalized amplitude of the BC last AP resulting from 100 Hz train stimulation. The acquisition protocol is described in Fig. S3. We wanted to test for fluctuations of the AP amplitude in each train related to the presence of the neuromodulators. We normalized the amplitude of the 50th AP to that of the first AP of the train and compared the normalized amplitude between control – 5-HT and control – NE. For both experiments we obtained consequential recordings from 5 cells each. A, Evaluating the effect of 5-HT on the amplitude of the last AP, control – black, 5-HT cyan, *p=0.7024*. B Evaluating the effect of NE on the amplitude of the last AP, control – black, NE – orange, *p=0.3532*


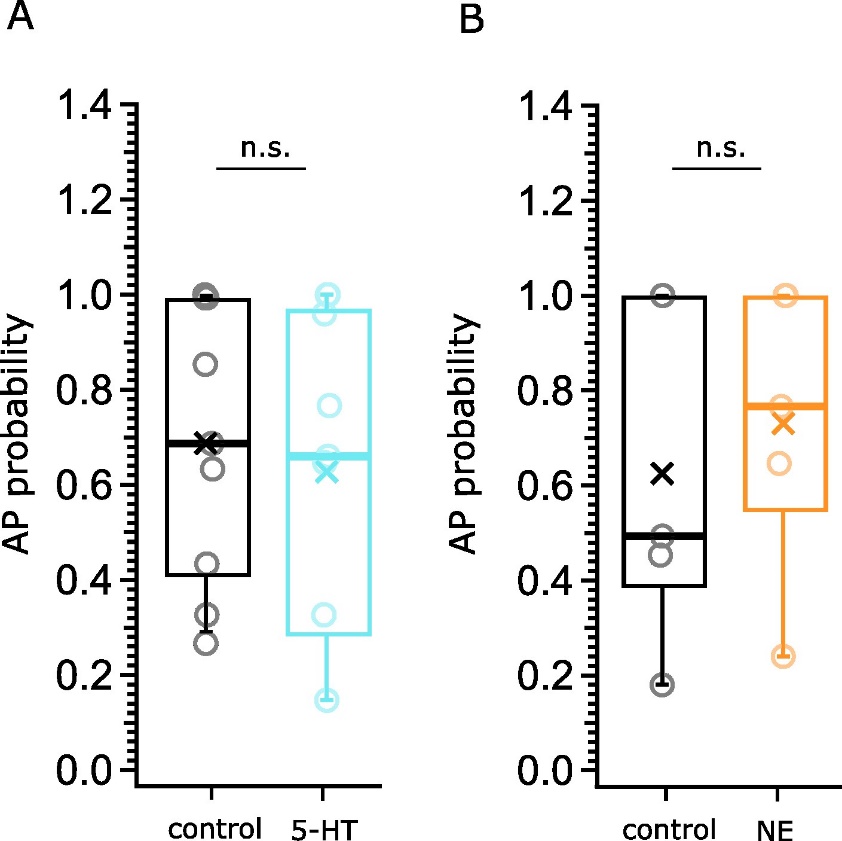


Figure S6 BC action potential (AP) probability at 200 Hz. The acquisition protocol is described in Fig. S3. At lower frequencies the BCs that we studied did not display failures during the trains. APs were normalized by the 1 AP. The AP probability was calculated as the number of AP divided by the number of stimuli (50) that the afferent fibers were subjected to. A, Evaluating the effects of 5-HT on the BC AP probability, control in black, 5-HT in cyan, N=9 cells, *p=0.70*; B, Evaluating the effects of NE on the BC AP probability, control in black, NE in orange, N=5 cells, p=0.63. The data is represented as box plots with minimum, first quartile, median, third quartile, and maximum, the cross represents the mean. n.s. – non-significant

Table S7 Comparison of parameters of trains of EPSCs at 100Hz and 200 Hz from the control and NE datasets measured at 1.3 mM Ca^2+^. N=4 cells from 4 animals for the 100 Hz recordings, n=3 cells from 3 animals for the 200 Hz recordings

| Frequency | parameter | control | NE | p value |
| --- | --- | --- | --- | --- |
| 100 Hz | Pvr_(EQ)_ | 0.09±0.01 | 0.10±0.02 | 0.88 |
|  | RRP_(EQ)_, SVs | 202.57±80.82 | 195.10±82.16 | 0.96 |
|  | PPR | 1.49±0.25 | 1.19±0.11 | 0.37 |
|  | τ of depression, ms | 68.04±7.72 | 77.15±21.77 | 0.75 |
| 200 Hz | Pvr_(EQ)_ | 0.15±0.03 | 0.08±0.02 | 0.30 |
|  | RRP_(EQ)_, SVs | 175.42±67.43 | 333.47±109.85 | 0.37 |
|  | PPR | 1.84±0.33 | 1.95±0.06 | 0.7 |
|  | τ of depression, ms | 44.37±8.87 | 48.32±13.01 | 0.85 |

Table S8 Comparison of parameters of trains of EPSCs at 100Hz and 200 Hz from the control and NE datasets measured right before and after NE application. N=6 cells from 4 animals

| frequency | parameter | control | NE | p value |
| --- | --- | --- | --- | --- |
| 100 Hz | Pvr_(EQ)_ | 0.15±0.01 | 0.10±0.01 | 0.05 |
|  | RRP_(EQ)_, SVs | 404.28±69.65 | 340.19±75.37 | 0.09 |
|  | PPR | 1.12±0.07 | 0.94±0.05 | 0.58 |
|  | τ of depression, ms | 39.64±5.59 | 47.03±7.44 | 0.36 |
| 200 Hz | Pvr_(EQ)_ | 0.16±0.03 | 0.12±0.02 | 0.27 |
|  | RRP_(EQ)_, SVs | 340.74±48.64 | 333.39±37.65 | 0.92 |
|  | PPR | 1.12±0.09 | 1.10±0.08 | 0.92 |
|  | τ of depression, ms | 35.04±11.09 | 25.37±3.86 | 0.44 |
